# Red-emissive thymine-functionalized carbon nanodots potentiate minocycline antibacterial activity at subtherapeutic doses

**DOI:** 10.1039/d6na00753h

**Published:** 2026-11-04

**Authors:** Océane Seivert, Yuta Takano, Junyue Qu, Yukari Maeda, Kiwamu Sakaguchi, Atsuro Yokoyama, Alberto Bianco, Eri Hirata

**Affiliations:** a CNRS, Immunology, Immunopathology and Therapeutic Chemistry, UPR 3572, University of Strasbourg, ISIS 67000 Strasbourg France a.bianco@ibmc-cnrs.unistra.fr; b Research Institute for Electronic Science, Hokkaido University Kita-20, Nishi-10 Sapporo 001-0020 Japan tak@es.hokudai.ac.jp; c Graduate School of Environmental Science, Hokkaido University N10, W5 Sapporo 060-0810 Japan; d Department of Oral Functional Prosthodontics, Faculty of Dental Medicine and Graduate School of Dental Medicine, Hokkaido University Sapporo 060-8586 Japan erieri@den.hokudai.ac.jp

## Abstract

Minocycline (MC), a second-generation tetracycline antibiotic, is commonly used to treat a broad range of bacterial infections, including periodontal infections. However, local application requires high concentrations, leading to a risk of cytotoxic effects on bone-forming cells and likely promoting resistance. Nanocarriers offer a means of concentrating MC at the site of application, yet their use depends on the reliable drug retention, which is governed by the drug–surface interaction. Here, red-emissive carbon nanodots (CNDs) were functionalized with a thymine-containing ligand (CND-L1), a nucleobase whose consecutive hydrogen-bond donor and acceptor sites are complementary to those of MC, enabling drug binding through a defined set of hydrogen bonds instead of by non-specific adsorption to the bare surface. The complexation efficiencies of both CNDs were quantified, and the resulting complexes were tested against *Aggregatibacter actinomycetemcomitans* (*A.a.*). While MC alone and CNDs/MC were inactive at a subtherapeutic concentration, approximately 13-fold below that delivered by commercial local formulations, CND-L1/MC reduced bacterial survival to 75%. The intrinsic red emission of the CNDs served as a label-free readout for confocal microscopy and quantitative flow cytometry, which revealed that CND-L1/MC associated approximately 4-fold more efficiently with *A.a.* cells than CNDs/MC, indicating that L1 modification preserves the complex-bacteria association under MC loading. All conjugates maintained MC3T3-E1 osteoblasts viability above 70%. CND-L1 therefore rendered MC active at a concentration at which the free drug had no effect.

## Abstract 2

Thymine-functionalized red-emissive carbon nanodots bind minocycline through hydrogen bonding, enabling localized delivery and boosting its antibacterial activity against the peri-implantitis pathogen *A. actinomycetemcomitans* at subtherapeutic doses.

## Graphical abstract

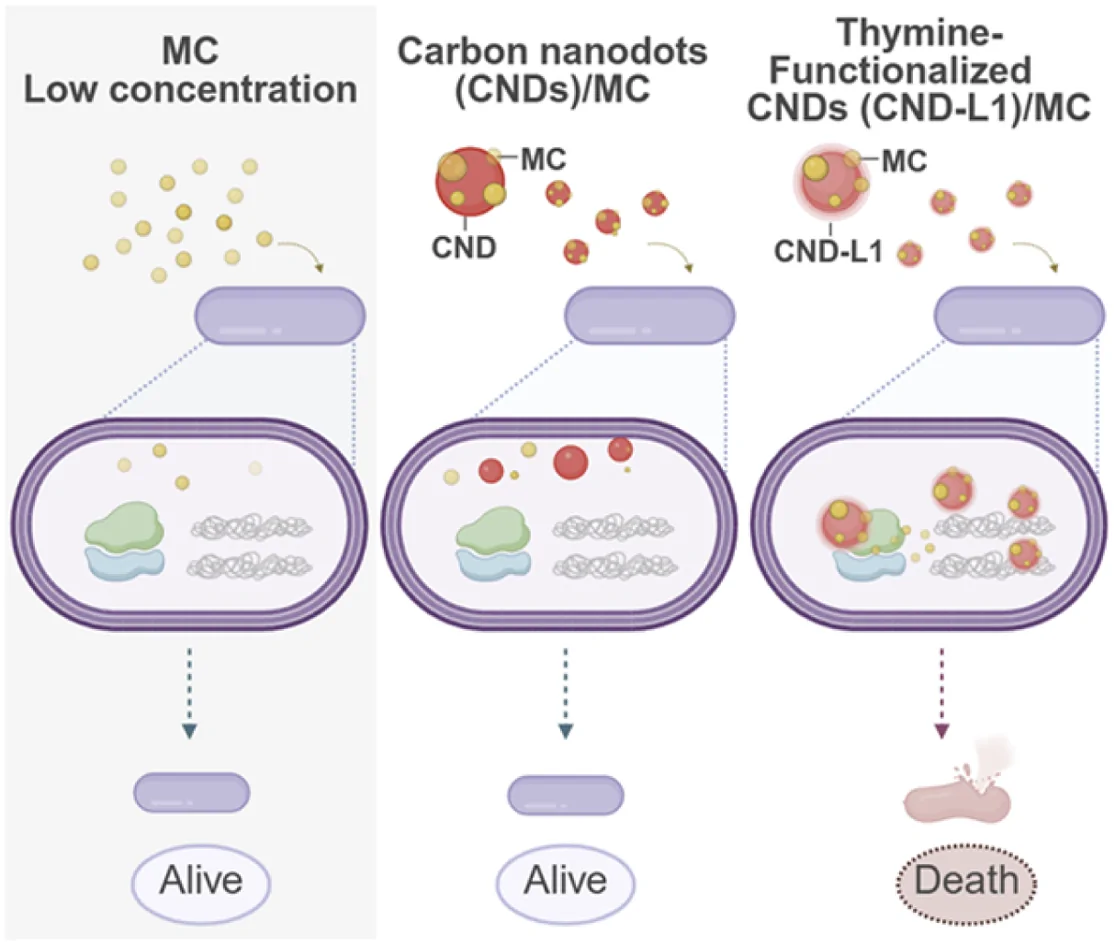

## 1 Introduction

Minocycline (MC), a second-generation tetracycline antibiotic, is active against a broad range of Gram-positive and Gram-negative bacteria. It also possesses anti-inflammatory properties, including inhibition of matrix metalloproteinases and modulation of host immune responses.^1^ Where an infection is confined to an accessible site, MC can be applied locally rather than systemically. The oral cavity is a prime example: periodontal and peri-implant sites are continuously exposed to bacteria, yet they are directly accessible, which makes local application both practical and effective. Controlled-release gels and microspheres containing MC have accordingly demonstrated clinical efficacy at these sites.^2,3^ Such formulations nonetheless face persistent limitations: burst release kinetics, short residence times at the infection site, and the need for relatively high antibiotic concentrations that may contribute to resistance development and disrupt the local microbiota.^4–6^ Moreover, high-dose tetracyclines can exert cytotoxic effects on the surrounding tissues.^7,8^

Around a dental implant, for example, this matters because the device is in direct contact with the surrounding bone, and its stability depends on the integrity of that contact, a state known as osseointegration. An antibacterial treatment that damages bone-forming cells would therefore compromise the outcome rather than exerting a protective action. The implant surface adds a second difficulty: it is deliberately rough, promoting osseointegration, but the micro- and nanoscale topography also provides additional area for bacterial attachment and shelters organisms in recesses, a condition that conventional formulations are not able to easily reach.^9^ A carrier able to complex an antibiotic and to reach these features is therefore of interest, and coating the implant surface with such a carrier is one of the promising strategies to prevent or treat bacterial infections.

Reaching the surface features described above requires a carrier of nanoscale dimensions. Size alone, however, is not sufficient: whether the carrier can hold and release the drug where it is needed depends on how the drug is bound to its surface. Carbon nanodots (CNDs) are well suited to address this question. Since their serendipitous discovery in 2004 during the purification of carbon nanotubes by electrophoresis, CNDs have emerged as a novel class of carbon-based nanomaterial attracting significant scientific interest.^10^ These new carbon-based nanomaterials exhibit unique physicochemical properties, including tunable fluorescence emission and good photostability. Such properties make CNDs highly promising for a wide range of biomedical applications, and they have already been explored in several therapeutic contexts.^11–14^ CNDs can be prepared through hydrothermal carbonization of small organic molecules or polymers,^15^ and the wide range of possible precursors gives access to diverse surface functionalities available for post-synthesis modification. The surface chemistry of a CND can therefore be designed to bind a drug in an appropriate manner, rather than relying on a non-specific and non-controlled adsorption to the bare surface of the dots. In this work, we used red-emissive CNDs, previously developed in our group, which combine several interesting features: (i) a high density of carboxylic acid groups on their surface, allowing to graft a drug-binding ligand directly by amide coupling without prior modification; (ii) an intrinsic red fluorescence emission, permitting to follow and quantify the association of these carriers with the bacterial cells by confocal microscopy and by flow cytometry, respectively, without an additional fluorescent labelling that could instead modify the surface chemistry; (iii) and a size in the nanometer scale (5 to 10 nm) (Fig. 2a).

In this study, we prepared minocycline-loaded CNDs with two distinct surface chemistries: direct adsorption of the drug onto the surface of bare CNDs, and a rationally designed conjugate in which a ligand (L1) was incorporated to enable stable hydrogen-bond interactions with the antibiotic (Fig. 1). The nucleobase thymine, which presents multiple consecutive hydrogen-bond donor and acceptor sites, was selected as a suitable candidate to interact with MC. *Aggregatibacter actinomycetemcomitans* (*A.a.*) was used as a recognized periodontal pathogen suitable for the comparative screening of carrier formulations.^16,17^ Comparing the two carriers allowed us to ask whether the mode of drug binding, and not the carrier alone, determines how much MC is loaded, how well it is retained, and whether it remains active at subtherapeutic doses. Because the two carriers share the same carbon core and differ only in their surface chemistry, this design isolates the contribution of the drug-binding mode, which a comparison between carriers of different chemical nature would not allow.

**Fig. 1:**
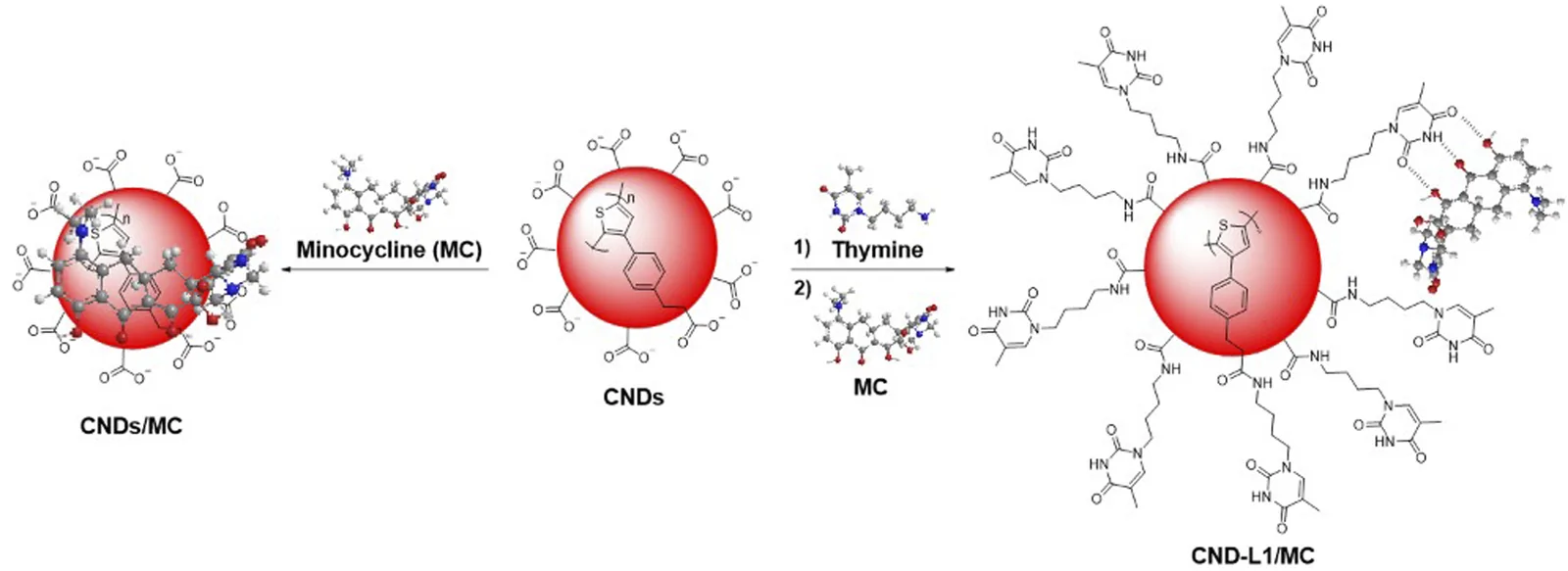
Schematic representation of minocycline complexation with bare CNDs (left) and CND-L1 (right).

## 2 Experimental section

### 2.1 Materials

All chemicals used in the experiments were obtained from commercial sources as analytical reagents and used as received. 3-(4-Bromophenyl)propionic acid, tetrakis(triphenylphosphine)palladium(0), iron(iii) chloride, *N*-(4-bromobutyl)phthalimide, hydrazine monohydrate, *N*-hydroxysuccinimide (NHS), agarose and dihydrorhodamine 123 (DHR123) were purchased from Sigma-Aldrich. Benzyl bromide and thymine were obtained from Acros Organics. 1-Ethyl-3-(3-dimethylaminopropyl)carbodiimide hydrochloride (EDC × HCl) was obtained from Alfa Aesar. 3-Thiophenylboronic acid was purchased from Fluorochem Ltd. 1-Bromobutane, Lambda DNA, and Gene Ruler (DNA Ladder mix) were obtained from Thermo Scientific. For the chemical analyses, minocycline hydrochloride was purchased from Thermo Fisher Scientific while for the biological analyses, it was obtained from FUJIFILM Wako. The dialysis was performed using membranes MWCO 1 kDa from Spectrum Laboratories Inc. Millipore Milli-Q^®^ water with a resistivity of 18.2 MΩ was used in all experiments.

### 2.2 Methods

UV-vis spectra were recorded using a VARIAN 5000 spectrometer at a scan rate of 600 nm min^−1^ between 200 and 800 nm. Fluorescence spectra were recorded using a Fluorolog FL3-22 spectrometer with a xenon 450 W lamp at a scan rate of 1000 nm min^−1^ between 500 and 800 nm. X-ray photoelectron spectroscopy was conducted using a multiprobe system (Thermo Scientific) equipped with an Al Kα source (1486 eV) at a pressure of 10^−8^–10^−9^ mbar in the main chamber. The X-ray spot size was settled at 400 µm. Survey spectra were recorded as a result of 20 scans with a pass energy of 200.00 eV and a step size of 1 eV, while high-resolution spectra were an average of 30 scans with a pass energy of 50.00 eV and a step size of 0.1 eV. All spectra were charge-corrected by referencing the C–C/C=C component of the C 1s spectrum to 284.8 eV and fitted using Thermo Scientific Avantage software after smart background subtraction. FT-IR spectra were recorded using a Thermo Fisher FT-IR iS50 spectrophotometer in ATR mode with a diamond crystal, over the range 4000–400 cm^−1^, at a spectral resolution of 4 cm^−1^, with 64 scans averaged per spectrum. Thermogravimetric analyses were performed using a TGA Q500 TA instrument with a heating rate of 10 °C min^−1^ from room temperature to 800 °C under N_2_ with a flow rate of 50 mL min^−1^ using platinum pans. Dynamic light scattering and zeta-potential measurements were performed using a Zetasizer Lab Blue instrument (Malvern Panalytical) with a 4 mW He–Ne 632.8 nm laser with carbon dots dispersed in Milli-Q^®^ water at a concentration of 25 µg mL^−1^. ^1^H and ^13^C NMR spectra were recorded at 25 °C on Bruker AV500 instrument operating at 500 MHz for ^1^H NMR and 125 MHz for ^13^C NMR. Tetramethylsilane or residual solvent peaks were used as internal reference. The peak values were obtained as ppm (*δ*) and referenced to the solvent. The resonance multiplicity is indicated as s (singlet), d (doublet), t (triplet), dd (doublet of doublet), and m (multiplet). Transmission electron microscopy images were collected on a Hitachi H 7500 instrument equipped with an AMT Hamamatsu digital camera and observed at 80 kV. The samples are deposited on 3.05 mm diameter 200-mesh formvar/carbon-coated copper grids. Circular dichroism spectra were recorded on a JASCO J-1700 spectropolarimeter under constant nitrogen purge. The CNDs or CNDs/DNA complexes were dispersed in Milli-Q^®^ water and measured in a 2 mm path-length quartz cuvette at 20 °C, with temperature controlled by a PTC-510 Peltier cell holder. Spectra were collected from 350 to 220 nm in continuous scanning mode at 100 nm min^−1^, with a bandwidth of 1.0 nm, a data pitch of 1 nm and a digital integration time of 1 s. Each spectrum is the average of 4 accumulations, and the spectrum of the solvent recorded under identical conditions was subtracted.

### 2.3 Synthesis of carbon nanodots

Red-emissive carbon nanodots were prepared as reported previously by our team.^16^ Briefly, CNDs were prepared through a five-step synthetic pathway starting from the protection of 3-(4-bromophenyl)propionic acid (96%) (compound 1), followed by a functionalization with 3-thiophene boronic acid using a Suzuki–Miyaura cross-coupling reaction (85%) (compound 2). Finally, deprotection of the benzyl group (81%) (compound 3), and an oxidative polymerization using FeCl_3_ were performed to generate the polymer polythiophene phenylpropionic acid (PPA). Water-dispersible CNDs were obtained by hydrothermal treatment in 1 M NaOH solution at 200 °C for 10 h. The solution was then filtered through 0.22 µm membranes (Millipores, PES) and the filtrate was dialyzed against Milli-Q^®^ water for 1 week (MWCO 1 kDa, water changed every morning, around 5 L). The solution is finally lyophilized to obtain CNDs as a dark red powder.

### 2.4 Synthesis of 1-(2-phthalimidobutyl)thymine (4)^18^

To a solution of thymine (2.00 g, 15.9 mmol) and K_2_CO_3_ (1.95 g, 14.13 mmol) in DMF (100 mL), *N*-(4-bromobutyl)phthalimide (1.49 g, 5.29 mmol) was added and the white mixture was stirred overnight at RT. The precipitate was filtered, and the filtrate was concentrated under reduced pressure. The whitish oil was dissolved in DCM (100 mL) and extracted with water (100 mL). The aqueous layer was extracted another time with DCM, and the organic phase was concentrated under reduced pressure. The yellowish oil was then dissolved in 3 mL of DCM and crystallized with cyclohexane to afford the desired compound 4 as a white powder (650 mg, 53%). ^1^H NMR (500 MHz, DMF-*d*_7_): *δ* 11.16 (1H, s), 7.89 (4H, m), 7.59 (1H, *J* = 1.2 Hz, q), 3.73 (4H, m), 1.79 (3H, *J* = 1.2 Hz, d) 1.70 (4H, *J* = 3.0 Hz, m). ^13^C NMR (125 MHz, DMF-*d*_7_): *δ* 206, 168.2, 165.1, 164.4, 151.3, 141.5, 137.8, 136.4, 132.1, 123.0, 108.11, 79.3, 47.1, 37.1, 26.4, 25.3, 12.1.

### 2.5 Synthesis of 1-(2-aminobutyl)thymine (ligand L1)

To a solution of compound 4 (100 mg, 0.305 mmol) in EtOH (20 mL), hydrazine monohydrate (0.1 mL, 1.22 mmol) was added, and the mixture was stirred overnight at reflux. The solvent was removed, the powder was dissolved in HCl (50 mL, 5%) and extracted with ether (2 × 50 mL). The yellowish powder was recrystallized in MeOH and ether was used as a counter solvent to give the desired compound L1 as a white powder (58 mg, 97%). ^1^H NMR (500 MHz, DMSO-*d*_6_): *δ* 11.23 (1H, s), 8.05 (2H, s), 7.59 (1H, *J* = 1.2Hz, q), 3.64 (2H, *J* = 6.7 Hz, t), 2.77 (2H, *J* = 6.7 Hz, q), 1.75 (3H, *J* = 1.1 Hz, d), 1.70–1.46 (4H, m). ^13^C NMR (125 MHz, DMSO-*d*_6_): *δ* 163.7, 150.6, 139.1, 108.5, 53.4, 43.8, 25.7, 22.2, 12.6.

### 2.6 Synthesis of CND-L1

A suspension of CNDs (5 mg) in Milli-Q^®^ water (10 mL) was sonicated in an iced bath for 10 min. NHS (20 mg), EDC × HCl (64 mg) and triethylamine (48 µL) were added, and the mixture was stirred at 0 °C for 4 h. A solution of ligand L1 (15 mg) in DMSO (0.5 mL) was added dropwise and the resulting solution is allowed to go back to RT and stirred for 20 h. The mixture was dialyzed against Milli-Q^®^ water for 2 days (MWCO 1 kDa, 3 L, water changed every morning and evening). After all treatment, a red solution of CND-L1 was obtained.

### 2.7 Synthesis of 1-butylthymine (5)^19^

To a solution of thymine (500 mg, 3.96 mmol) in DMF (40 mL), 1-bromobutane (142 µL, 1.32 mmol), K_2_CO_3_ (456 mg, 3.3 mmol) and KI (0.11 mg, 0.6 mmol) were added, and the resulting solution was heated at 40 °C overnight. The white precipitate was then filtered, and the filtrate was concentrated under reduced pressure. The crude yellowish powder was dissolved in DCM (20 mL) and extracted with water (3 × 20 mL). The organic layer was concentrated under reduced pressure, and the resulting powder was dried under vacuum to give the desired product 5 as a white greasy solid (180 mg, 75%). ^1^H NMR (500 MHz, 0.5 mL CDCl_3_ + 50 µL of DMF-*d*_7_): *δ* 8.89 (s, 1H), 6.97 (q, *J* = 1.2 Hz, 1H), 3.75–3.63 (m, 2H), 1.92 (s, 2H), 1.68–1.60 (m, 2H), 1.48–1.21 (m, 3H), 0.99–0.93 (m, 3H). ^13^C NMR (125 MHz, 0.5 mL CDCl_3_ + 50 µL of DMF-*d*_7_): *δ* 164.3, 150.9, 141.5, 108.3, 54.9, 30.5, 19.6, 13.7.

### 2.8 Complexation with minocycline

#### 2.8.1 Compound 5/MC

Compound 5 and minocycline were solubilized separately in a mixture of CDCl_3_/DMF-*d*_7_ (10 : 1) to reach a volume of 275 µL for each solution. The solution of minocycline (275 µL) was added to compound 5 solution (275 µL) in a 1 : 1 mol mol^−1^ ratio. The resulting mixture was stirred for 5 min before being transferred into an NMR tube. ^1^H NMR spectrum was recorded and the shift of the proton of interest was studied.

#### 2.8.2 CNDs/MC

To a solution of CNDs (1 mL) at a concentration of 296 µg mL^−1^ in Milli-Q^®^ water, 4 µL of the previously prepared MC solution was added dropwise while sonicating and the resulting solution was stirred at RT for 24 h. The solution was used without any further purifications. This procedure allowed to obtain a solution of CNDs/MC complex with 40 µg of MC complexed to 300 µg of CNDs.

#### 2.8.3 CND-L1/MC

To a solution CND-L1 (1 mL) at a concentration of 296 µg mL^−1^ in Milli-Q^®^ water, 10 µL of the previously prepared MC solution was added dropwise while sonicating and the resulting solution was stirred at RT for 24 h. The solution was then centrifuged for 30 min at 4 °C with a speed of 25 000 g. The CND-L1/MC complex was filtered using a Millipore membrane filter with a pore size of 50 nm and CND-L1/MC was washed 2 times with 100 µL of Milli-Q^®^ water. The complex was then dispersed again in 1 mL of Milli-Q^®^ water. This procedure allowed to obtain a solution of CND-L1/MC complex with 40 µg of MC complexed to 300 µg of CND-L1.

Note: for Sections 2.8.2 and 2.8.3, a stock solution of 1 mg of MC in 100 µL of Milli-Q^®^ water was freshly prepared.

### 2.9 Bacterial culture and antibacterial assays

*Aggregatibacter actinomycetemcomitans* (*A.a.*) was cultured under standard conditions and diluted to a concentration of 1.6 × 10^4^ CFU mL^−1^. The test formulations were prepared as follows: phosphate-buffered saline (PBS) as a control (CTRL), minocycline (MC) at 32.4 µg mL^−1^, CNDs at 240 µg mL^−1^, CND-L1 at 240 µg mL^−1^, CNDs/MC complex (240 µg mL^−1^ CNDs + 32.4 µg mL^−1^ MC), and CND-L1/MC complex (240 µg mL^−1^ CND-L1 + 32.4 µg mL^−1^ MC). For CNDs/MC and CND-L1/MC complexes, MC was pre-incubated with CNDs or CND-L1 according to the complexation protocol described in Section 2.8.

These concentrations correspond to the maximum MC loading achievable with the available CND material (Section 3.2), and the dose-response experiment in Fig. S7 confirms that free MC applied at this concentration does not reduce bacterial survival. The inoculum was chosen to give a countable number of colonies after plating, since bacterial survival was quantified by colony counting rather than by broth microdilution. It is therefore lower than that used in standard susceptibility testing, and the concentrations reported do not correspond to minimum inhibitory concentrations. For antibacterial testing, 100 µL of each sample was mixed with 15 µL of bacterial suspension (1.6 × 10^4^ CFU mL^−1^) in a 96-well microplate and incubated for 5 min at room temperature. The samples are aqueous dispersions. Addition of 10× PBS at a sample to 10× PBS ratio of 10 : 1 brings them to 1× PBS, so that the suspension is isotonic and buffered before contact with the bacteria. The identical procedure was applied to the control group. This 1.1-fold dilution gave buffered stock concentrations of 29.5 µg mL^−1^ MC (stock samples) and 218.2 µg mL^−1^ CNDs or CND-L1. Mixing 100 µL of this buffered sample with 15 µL of bacterial suspension corresponds to a further 1.15-fold dilution, so that the final concentrations in the assay medium were 25.6 µg mL^−1^ for MC and 189.7 µg mL^−1^ for CNDs or CND-L1. The bacterial suspensions were then seeded onto agar plates and incubated at 37 °C under microaerophilic conditions (5% CO_2_, 10% H_2_, 85% N_2_) for 48 h. Colony-forming units (CFUs) were manually enumerated, and bacterial survival rates were calculated relative to the CTRL group (designated as 100%). Four independent experiments were performed (*n* = 4 plates per treatment group per experiment).

To validate the MC concentration range, a dose-response experiment was conducted using MC alone at three concentrations: 29.4 µg mL^−1^ (1×, designated as MC), 295 µg mL^−1^ (10×, designated as MC10), and 2945 µg mL^−1^ (100×, designated as MC100). Following 10× PBS dilution, the final concentrations in the assay medium were 25.6, 256, and 2560 µg mL^−1^, respectively. Bacterial cultures were processed as described above.

### 2.10 Confocal laser scanning microscopy imaging

*A.a.* was incubated with CNDs, CND-L1, CNDs/MC, and CND-L1/MC (218 µg mL^−1^ of carbon nanodots and 29 µg mL^−1^ of MC for the complexes) for 5 min at RT, followed by washing with PBS. The bacteria were viable during this incubation; fixation was carried out only afterwards. A short incubation time of 5 min was selected to verify the quick association of the complexes with the bacterial surface, before the antibiotic release and the resulting effects on the bacteria likely affecting measurement. The cells were fixed with 4% (w/v) paraformaldehyde for 10 min at room temperature and washed three times with PBS. The fixed bacteria were stained with Hoechst 33342 (final concentration: 1.0 µg mL^−1^) for 15 min at RT in the dark. After washing away the staining solution with PBS, the cell suspensions of PBS were transferred to a glass-bottom dish (AGC Techno Glass Co., Japan) coated with poly-d-lysine (Aldrich, USA). Confocal laser scanning microscopy was performed on a Nikon A1 system (Ti2 body) with a Plan Apo VC 100× oil-immersion objective (NA 1.4). Hoechst 33342 was excited at 403 nm (3% laser power, ∼0.2 mW at the sample; PMT 80 V), with emission collected at 420–480 nm to visualize the bacterial nucleoids. CND autofluorescence was excited at 489 nm (100% laser power, ∼8.1 mW at the sample; PMT 100 V), with emission collected at 600–720 nm. The pinhole diameter was 135.4 µm. Images (512 × 512 pixels, 12-bit, 82.8 nm per pixel) were single optical sections (no *z*-stack), acquired sequentially with 16× line averaging to minimize spectral crosstalk between channels. Because the bacteria were fixed but adhered to the substrate only through the poly-d-lysine coating, non-adhered cells lying outside the single focal plane can appear in the bright-field channel without a matching fluorescence signal (Section 3.4.1). Merged images combining Hoechst, CNDs, and differential interference contrast (DIC) channels were generated to assess the spatial co-localization of CNDs with bacterial cells.

### 2.11 Flow cytometry analysis

Flow cytometry was used to quantify the association of CNDs and their MC complexes with *A.a*. cells. Viable *A.a.* (1.0 × 10^6^ CFU mL^−1^ final concentration, set by the event rate recommended for the instrument) was incubated with PBS (negative control), CNDs/MC, CND-L1/MC, or CND-L1/MC suspended in PBS without bacteria (no-bacteria control, included to verify that free aggregates do not fall within the bacterial gate). Both conjugates were used at 218 µg mL^−1^ of CNDs and 29 µg mL^−1^ of MC, corresponding to the same MC : CNDs mass ratio as in the other experiments. These concentrations are higher than those used in the antibacterial and cytotoxicity assays because the measurement relies on the intrinsic red emission of the CNDs and required to detect a signal above the bacterial autofluorescence. After 10 min at room temperature the suspensions were centrifuged and the cells were fixed with 4% (w/v) paraformaldehyde for 10 min. Fixation was therefore performed after exposure, so that the association reported here took place with viable bacteria. Samples were then stained with Hoechst 33342 (1 µg mL^−1^) for 15 min in the dark, washed twice with 0.22 µm-filtered PBS by centrifugation (4000×*g*, 5 min), and resuspended in filtered PBS for acquisition. Data were acquired on a BD FACSCanto II (BD Biosciences, USA) using a 405 nm laser with Pacific Blue (450/50 nm bandpass) for Hoechst detection and a 488 nm laser with the phycoerythrin (PE) channel (585/42 nm bandpass) for CND red autofluorescence. Single-bacteria events were gated as Hoechst-positive (Pacific Blue-H > 100). The median PE-H fluorescence intensity within the Hoechst-positive gate was used as the measure of CND association per bacterium. The experiment was performed in triplicate (*n* = 3 technical replicates per group). FCS files were processed in Python (fcsparser, NumPy, pandas) and statistical comparisons (Welch's *t*-test) were performed in GraphPad Prism 10 (GraphPad Software, USA).

### 2.12 ROS generation

The production of ROS by the carbon dots was evaluated using dihydrorhodamine 123 (DHR123). In a typical assay, 250 µL of CNDs or CND-L1 (at 25 µg mL^−1^) were added to 250 µL of an aqueous solution of DHR123 (at 20 nM). The resulting mixture was irradiated with either an 808 nm laser (1.2 W cm^−2^), or a 660 nm laser (0.1 W cm^−2^) for 0 to 10 min. As a control, the same procedure was performed without irradiation and with sample kept in the dark for 0 to 10 min. Every minute, the emission spectrum was recorded between 500 and 800 nm upon excitation at 488 nm. The fluorescence intensity at 534 nm was quantified after normalization to the carbon dots emission peak at 628 nm and subtraction of the emission signal of the carbon dots.

### 2.13 Cell culture and cytotoxicity assay

MC3T3-E1 murine osteoblast-like cells (ATCC) were cultured in α-minimum essential medium (α-MEM) supplemented with 10% (v/v) fetal bovine serum and 1% (v/v) penicillin–streptomycin at 37 °C in a humidified atmosphere containing 5% CO_2_. For the cytotoxicity assessment, cells were seeded at 2000 cells per well in 100 µL of medium in 96-well plates and incubated for 24 h to allow cell attachment. The different samples were prepared as follows: CTRL (PBS), MC (0.66 µg mL^−1^ final concentration), CNDs (5 µg mL^−1^ final concentration), CND-L1 (5 µg mL^−1^ final concentration), CNDs/MC, and CND-L1/MC complexes. As for the bacterial experiments, 10× PBS was added to the aqueous dispersions at a sample to 10× PBS ratio of 10 : 1 to bring them to 1× PBS before dilution. The buffered dispersions were then diluted in complete culture medium, and 100 µL of each diluted sample was added to 100 µL of medium containing the cells (200 µL final volume per well), so that the cells were maintained in complete culture medium for the whole duration of the experiment. The untreated control consisted of cells maintained in complete culture medium over the same period. Since MC is carried on the CNDs surface, the two concentrations cannot be set independently, and the CNDs concentration was chosen first. Cell viability was assessed at 4, 8, 24, and 48 h post-treatment using the WST-1 assay (Roche) following the manufacturer's instructions. Briefly, 20 µL of WST-1 reagent was added to each well, and the plates were incubated for 2 h at 37 °C. The absorbance was measured at 450 nm using a microplate reader with a reference wavelength of 690 nm. The background absorbance of cell-free wells containing each sample formulation was measured separately and subtracted from the corresponding experimental data. Cell viability was expressed as a percentage relative to that of the untreated control (CTRL) at each time point. Data are presented as mean ± standard deviation from three independent experiments, each performed with five wells per treatment group.

### 2.14 Statistical analysis

Statistical significance was assessed using one-way ANOVA, followed by Tukey's multiple comparison test (*P < 0.05, **P < 0.01, ***P < 0.001).

## 3 Results and discussion

### 3.1 Synthesis and characterization of carbon nanodots

Red-emissive CNDs used in this study were prepared following the synthesis that we reported previously.^20^ Briefly, CNDs were prepared *via* a hydrothermal treatment of polythiophene phenylpropionic acid in an alkaline solution (Scheme S1). The resulting carbon materials were subsequently covalently functionalized with a thymine derivative (ligand L1, Scheme S2 and Fig. 1). Ligand L1, designed with a terminal thymine moiety to enable complexation with minocycline, was synthesized from thymine and *N*-(4-bromobutyl)phthalimide followed by the deprotection of the phthalimide group using hydrazine monohydrate (Scheme S2). The functionalization of CNDs with ligand L1 was performed through amide coupling between the abundant surface carboxylic acids on CNDs and the terminal amine of L1, yielding the conjugate CND-L1.

Purification of the carbon nanodots (CNDs and CND-L1) is essential and critical, as inadequate cleaning remains a major challenge in CND research. Residual contaminants (precursors or unreacted species) can alter the properties and characteristics of CNDs, interfere with biological outcomes, induce toxicity, compromise biocompatibility, and generate false-positive results.^21^ Therefore, ensuring a high degree of purity is essential for reproducible and reliable data. Following the purification, both CNDs and CND-L1 were characterized using transmission electron microscopy (TEM), dynamic light scattering (DLS), zeta potential, Fourier-transform infrared spectroscopy (FT-IR), X-ray photoelectron spectroscopy (XPS), UV-vis and fluorescence spectroscopy, and thermogravimetric analysis (TGA).

The morphology and size of the two CNDs were examined by TEM (Fig. 2a and b). Pristine CNDs appeared well-dispersed, quasi-spherical nanoparticles with diameters of approximately 5 to 10 nm, while no aggregates were observed (Fig. 2a). In contrast, two populations coexisted in the CND-L1 sample, as shown by the two images in Fig. 2b, which were recorded on different areas of the same grid. The left image shows isolated nanodots with slightly larger diameters than pristine CNDs (15 to 20 nm), whereas the right image shows aggregated structures several hundred nanometres in size. This aggregation can be explained by intermolecular hydrogen bonding between the thymine moieties of neighbouring CND-L1 particles, together with the reduced electrostatic repulsion resulting from the consumption of surface carboxylic acid groups during the coupling with L1. These observations were further confirmed by DLS and zeta potential measurements. At neutral pH, CNDs (25 µg mL^−1^) displayed an average hydrodynamic diameter of 9.6 nm and a zeta potential of −35.1 mV, consistent with small, well-dispersed, and negatively charged nanoparticles. In contrast, CND-L1 exhibited a larger hydrodynamic diameter of 267 nm and a zeta potential of −17.5 mV, confirming its tendency to aggregate.

**Fig. 2:**
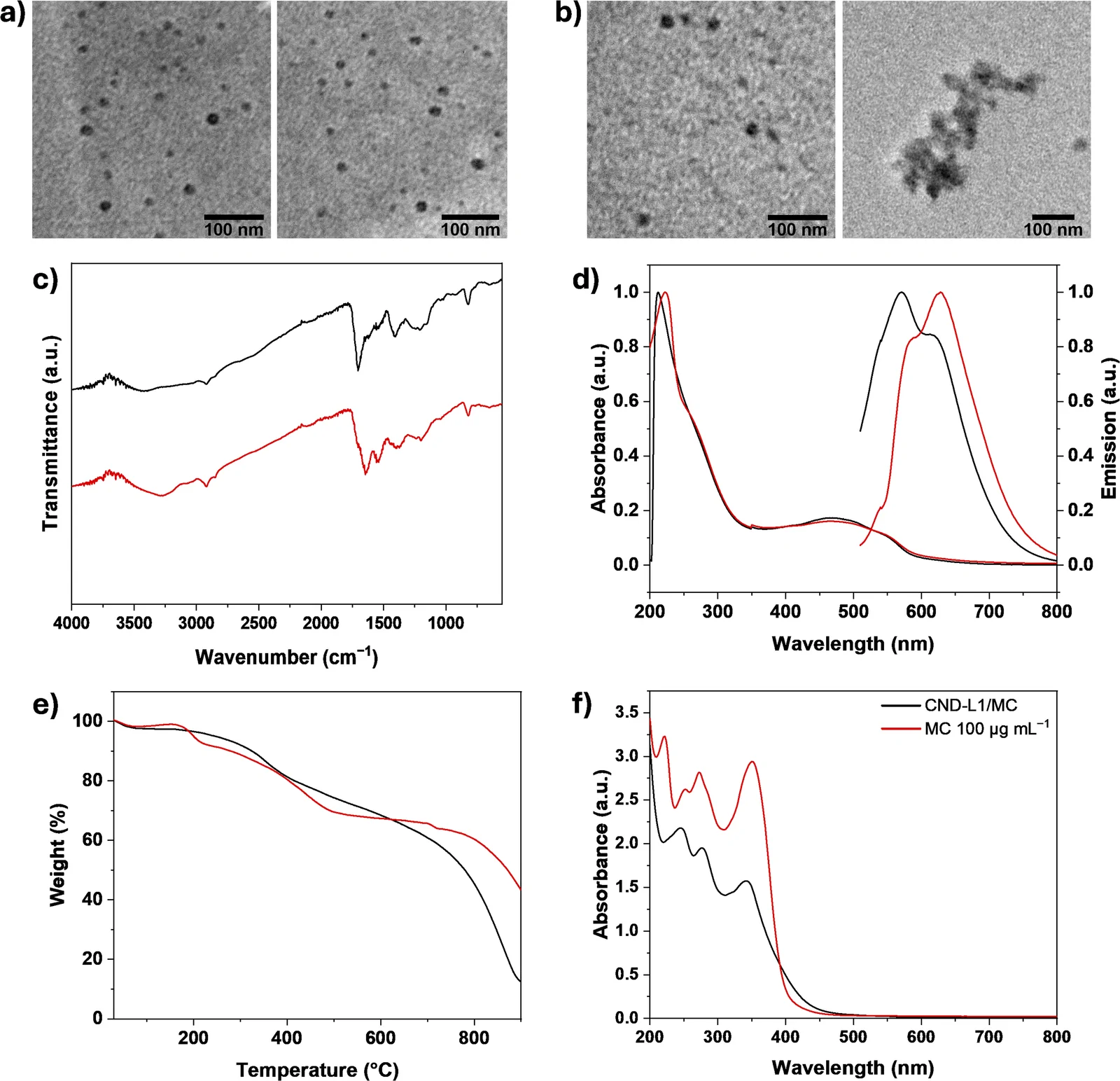
TEM images of (a) pristine CNDs and (b) CND-L1, showing isolated nanodots (left) and aggregated structures (right) recorded on different areas of the same grid, (c) FT-IR spectra of CNDs (black line) and CND-L1 (red line), (d) UV-vis absorption and fluorescence emission spectra of CNDs (black line) and CND-L1 (red line), (e) TGA curves of CNDs (black line) and CND-L1 (red line), and (f) UV-vis spectrum (black line) of the filtrate obtained after centrifuging the CND-L1/MC complex, compared with the UV-vis spectrum (red line) of MC at 100 µg mL^−1^.

These measurements show that CND-L1 is less negatively charged and has a greater tendency to aggregate than pristine CNDs. Two nonexclusive explanations are possible. First, the coupling reaction converts part of the surface carboxylic acid groups into amides, as supported by FT-IR and XPS. This would reduce the number of ionizable groups exposed to the solvent and the electrostatic repulsion between the particles, consistent with the less negative zeta potential. Second, the grafted thymine units could promote attractive interactions between neighbouring particles, through intermolecular hydrogen bonding and/or π-stacking between the nucleobases. The FT-IR spectra of CNDs and CND-L1 were recorded in the solid state at room temperature (RT) (Fig. 2c). Both spectra exhibited a weak band at 819 cm^−1^, corresponding to the C–S stretching vibration of the thiophene moiety, embedded in the core of the carbon nanodots. Compared to CNDs, the spectrum of CND-L1 displayed a shift to lower wavenumber of the carbonyl stretching band from 1702 cm^−1^ (carboxylic bond) to 1645 cm^−1^ (amide bond), along with the appearance of a new band at 3268 cm^−1^ assigned to N–H stretching vibrations, supporting the covalent modification of CNDs with ligand L1.

XPS was also employed to characterize the chemical compositions of CNDs and CND-L1 (Fig. 3 and 4). The XPS survey and high-resolution C 1s and O 1s spectra of CNDs displayed a similar profile to that previously reported.^20^ The survey spectrum of CNDs revealed the presence of 80% of C, 16% of O, and 4% of S. For CND-L1, the survey indicated 76% of C, 14% of O, 7% of S and an additional 4% of N, consistent with the successful introduction of the nitrogen-containing ligand L1. The high-resolution C 1s spectra of both materials were deconvoluted into five peaks at 284.66 (C–C/C=C), 285.19 (C–O/C–S), 286.13 (C–N), 286.99 (C=O), and 288.45 eV (COOH/π–π* satellite). For CND-L1, the high-resolution N 1s spectrum displayed two main deconvoluted peaks at 400.14 and 402.14 eV, attributed to the amide (N–C=O) and tertiary amine (N–R_3_) bonds, respectively.

**Fig. 3:**
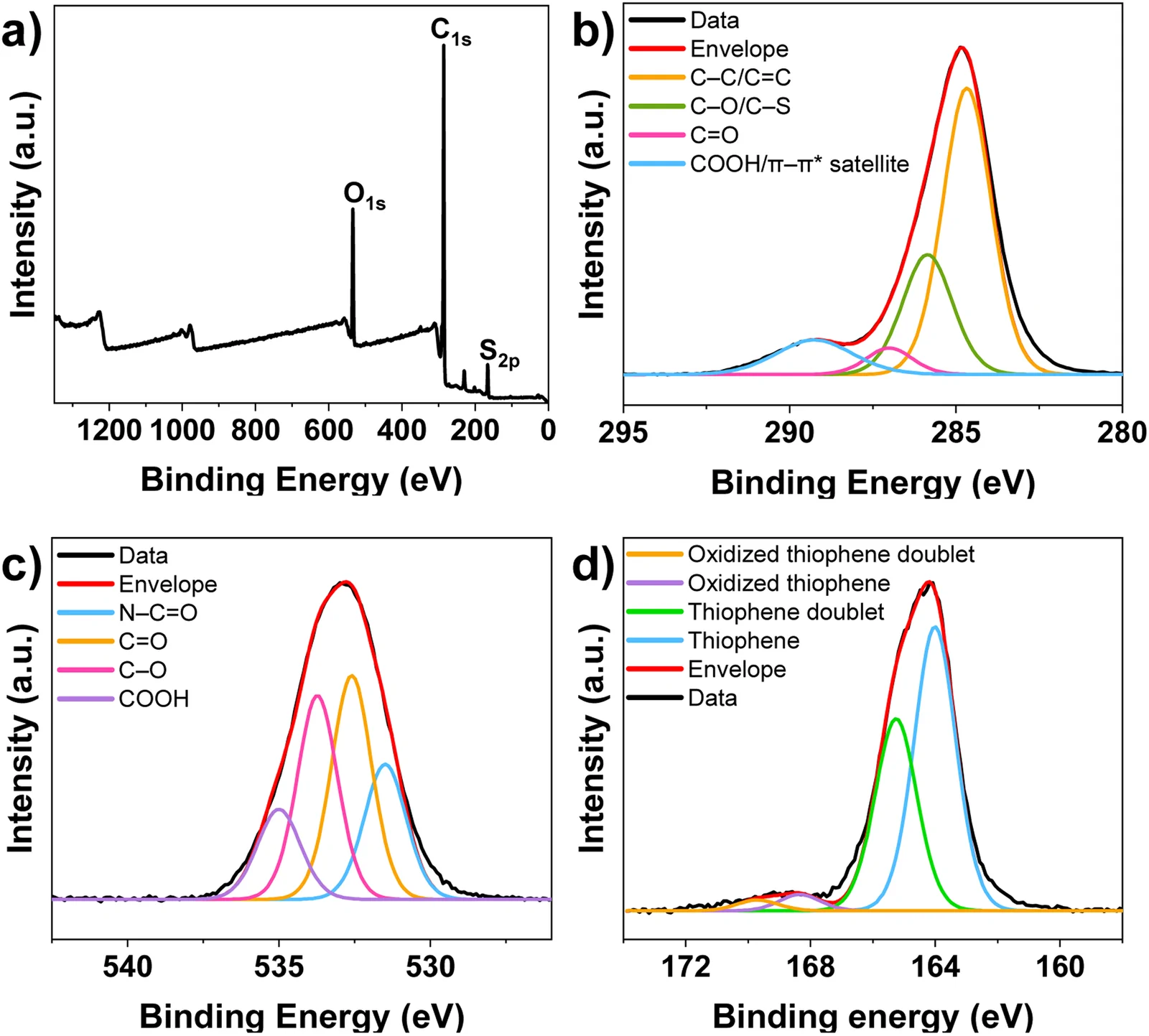
(a) XPS survey; (b) high-resolution C 1s; (c) high-resolution O 1s; and (d) high-resolution S 2p spectra of pristine CNDs.

**Fig. 4:**
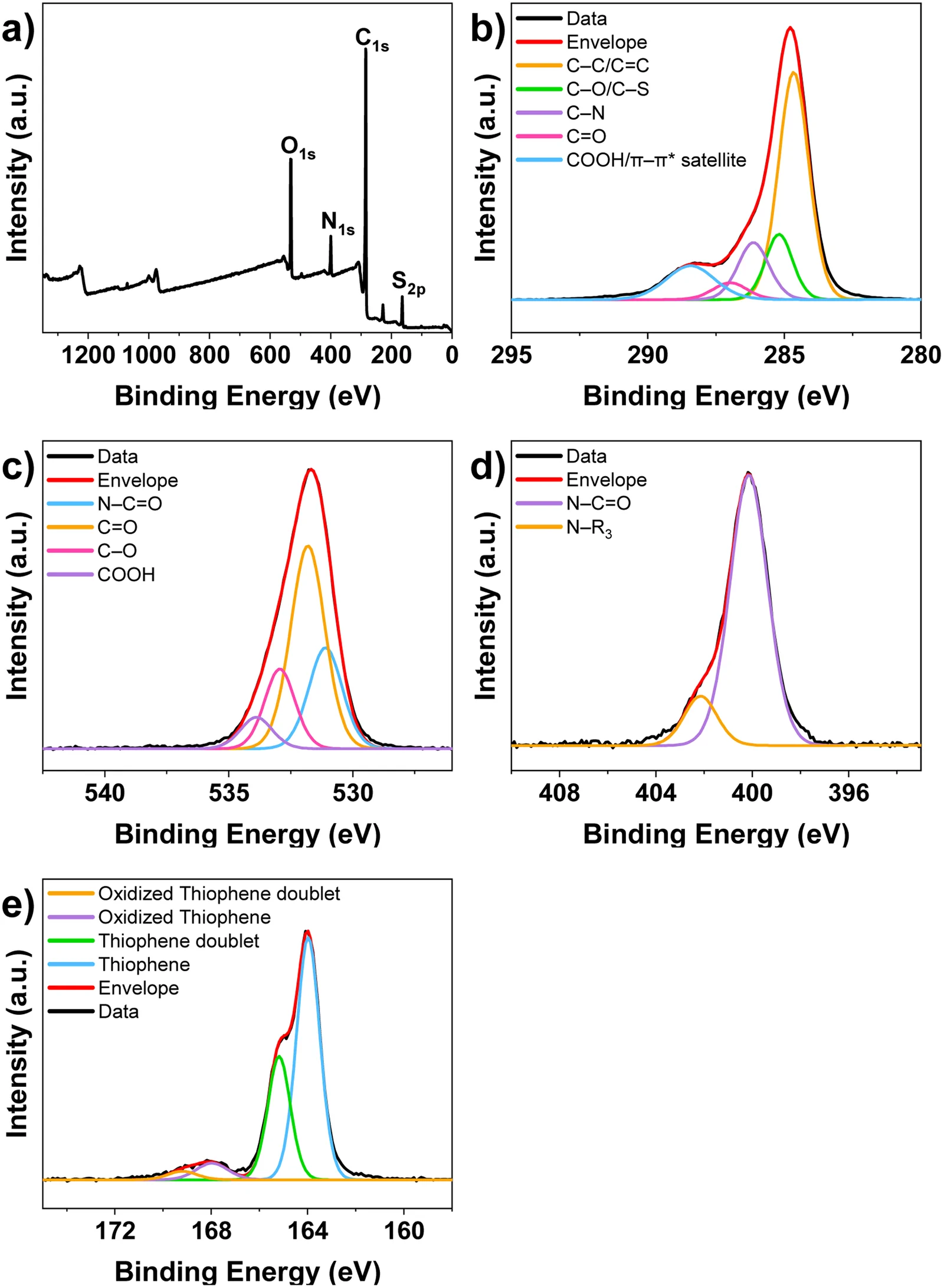
(a) XPS survey; (b) high-resolution C 1s; (c) high-resolution O 1s; (d) high resolution N 1s; (e) and high-resolution S 2p spectra of CND-L1.

The UV-vis and fluorescence spectra of CNDs and CND-L1 were recorded at a concentration of 25 µg mL^−1^ in Milli-Q^®^ water (Fig. 2d). Both carbon nanodots exhibit similar absorption profiles with the main band at 212 nm corresponding to the π–π* transitions and a shoulder around 263 nm attributed to the n–π* transitions, consistent with previous reported literature.^22^ Typically for carbon dots, two absorption bands are observed in the UV regions, with a maximum peak between 200 and 270 nm assigned to the π–π* transitions of aromatic C–C bonds and a shoulder present between 280–350 nm assigned to the n–π* transitions of C=O, C–O bonds, or other connected groups. The functional groups might also be partially responsible for the absorption in the UV-visible regions.^22^ In addition to the two UV bands, pristine CNDs presented an absorption broad band in the visible region at 488 nm with a shoulder at 540 nm. The fluorescence emission spectra were subsequently recorded between 500 and 800 nm with an excitation wavelength of 488 nm. The CNDs exhibited emission bands at 570 and 629 nm, whereas CND-L1 emitted at 590 and 629 nm. Notably, a red shift of 20 nm was observed for the first emission band upon functionalization. This red-shift is likely due to the introduction of thymine moieties on the CND surface, which promotes hydrogen bond interactions between neighboring particles and a slight tendency to aggregate, as confirmed by TEM and DLS analyses. It is well-established that the photoluminescence properties of CNDs are strongly governed by their surface states.^23,24^ In particular, several studies have shown that when these materials undergo aggregation, either through solvent-induced interactions or hydrogen-bond formation between surface groups, the resulting assemblies exhibited red-shifted emission due to the stabilization of lower-energy emissive states.^25–27^ This behavior is attributed to the electronic domain overlapping occurring within aggregated clusters, which enables through-space conjugation, expands the π-system, and narrows the band gap, inducing the observed red-shift in the emission spectrum.^28–31^

The thermal behaviors of CNDs and CND-L1 were evaluated using TGA (Fig. 2e). The TGA profile of CNDs displayed a two-steps thermal degradation, with a first weight loss at 300 °C corresponding to the thermal degradation of the surface groups and the transformation of less ordered carbon domain into more stable graphitic structure.^32^ This was followed by a second weight loss of 48% at 650 °C, attributed to the degradation of the carbon core of CNDs. In comparison, CND-L1 exhibited four decomposition steps at 150 °C (desorption of water), 225 and 500 °C (thermal degradation of the surface ligand and transformation into a graphitic structure), and 790 °C (degradation of the carbon core). Notably, CND-L1 demonstrated enhanced thermal stability, and the carbon core remained intact up to 790 °C. The increased number of degradation steps can be explained by the higher heterogeneous composition of CND-L1. In pristine CNDs, the surface consists mainly of carboxylic acid groups, which decompose within a single temperature range (decarboxylation around 300 °C). After grafting, the surface carries several components with different thermal stabilities. The first step at 150 °C corresponds to water retained by the hydrophilic, hydrogen-bonding thymine units. The step at 225 °C can be attributed to cleavage of the more labile part of the ligand, the alkyl linker and the amide bond. The step at 500 °C can be attributed to the thermal decomposition of the CNDs. The enhanced thermal stability of the carbon core may arise from several contributions. First, converting a large fraction of the surface carboxylic acids into amide bonds removes groups whose decarboxylation generates defects in the carbon framework at relatively low temperature. Secondly, the intermolecular hydrogen bonding between thymine moieties, which causes the aggregation of CND-L1 observed by TEM and DLS, produces a denser, cross-linked material in which heat and mass transfer are slowed down, and the release of volatile fragments is delayed. Similar increases in thermal stability after surface functionalization of carbon dots were previously reported.^33,34^

### 3.2 Complexation of minocycline to carbon nanodots

Minocycline is a broad-spectrum and anti-inflammatory antibiotic.^35^ It acts by inhibiting bacterial protein synthesis by targeting the 30S ribosomal subunit, and its high solubility and prolonged activity against both Gram-positive and Gram-negative bacteria make it especially suited for treating oral and maxillofacial infections.^36,37^ Compared with traditional broad-spectrum antibiotics, minocycline has stronger antibacterial activity, higher local concentration, and longer duration of action. However, systemic oral administration delivers only a small amount of the drug at the local site of infection.^38^ To overcome this limitation, local drug delivery systems (LDDS) were developed in the periodontal and peri-implant pocket.^39^ and minocycline has been widely utilized in these LDDS due to its broad-spectrum activity and sustainability.^40^ However, the overuse of antibiotics raises iatrogenic concerns, including the emergence of antibiotic-resistant bacteria and superinfections,^41^ highlighting the need for more efficient LDDS materials capable of achieving therapeutic effects at lower concentrations. In contrast, up to now, no studies have explored the complexation of minocycline with alternative carbon materials like CNDs, for the local treatment of peri-implantitis.

Minocycline was complexed to CNDs either through direct adsorption on the surface of the pristine CNDs or by hydrogen bonding on CND-L1 (Fig. 1), with the aim to use them in a local delivery approach. Direct adsorption at the surface of pristine CNDs provides weaker bonding of minocycline, leading to lower stability of the complex but allowing faster drug release. In contrast, H-bond complexation onto CND-L1 offers stronger stability and thus a slower and a better controlled drug release over time.

In order to elucidate the stability of the CND-L1/MC complex, the ability of the thymine moiety to form H-bonds with minocycline was assessed using ^1^H NMR and the model compound 5 (Scheme S3 and Fig. S1–S4). Compound 5 consists of a thymine derivative substituted with a simple alkyl chain at the N1 position of the pyrimidine ring, mimicking ligand L1 while minimizing potential additional interactions (likely due to the amino function of L1) with minocycline beside the specific H-bonding between the antibiotic and thymine moiety. A model compound was required because the interaction could not be studied directly using CND-L1 by ^1^H NMR. CND-L1 and CNDs, in general, are aqueous dispersible materials characterized by broad NMR signals. Consequently, the signals of the ligands grafted onto this type of nanoparticles will be also strongly broadened, preventing a sufficient resolution of the individual chemical shifts.^42^ Compound 5 reproduces the thymine unit of L1 in an aprotic medium in which H-bonding interactions can be easily observed. This study was also conducted in organic and aprotic solvents to minimize solvent competition that can disrupt the H-bonding interactions. The addition of protic solvents during ^1^H NMR titrations served as H-bond competitors to influence the chemical shifts of the NH or OH functions, free or involved into intermolecular H-bonds.

A mixture of CDCl_3_ and DMF-*d*_7_ (10 : 1 v/v) was used to minimize the competitive hydrogen bonding by dimethylformamide while ensuring good solubility for both compound 5 and MC (MC is poorly soluble in chloroform). The ^1^H NMR spectra of each compound were first recorded individually (Fig. S1 and S2). The proton assignments for minocycline was done according to previously reported data.^43^ The two hydroxyl groups of minocycline at positions C10 and C12 (see structures in Table 1), involved in the potential hydrogen bonding, have chemical shifts of 11.43 and 14.56 ppm, respectively, while the N3–H proton of compound 5 has a chemical shift of 8.89 ppm (Table 1, line 3).

**Table 1:** ^1^H-NMR chemical shifts of the protons potentially forming H-bonds in MC and compounds 5 (individually or in complex) in deuterated solvents

NMR solvents	Minocycline		Compound 5
	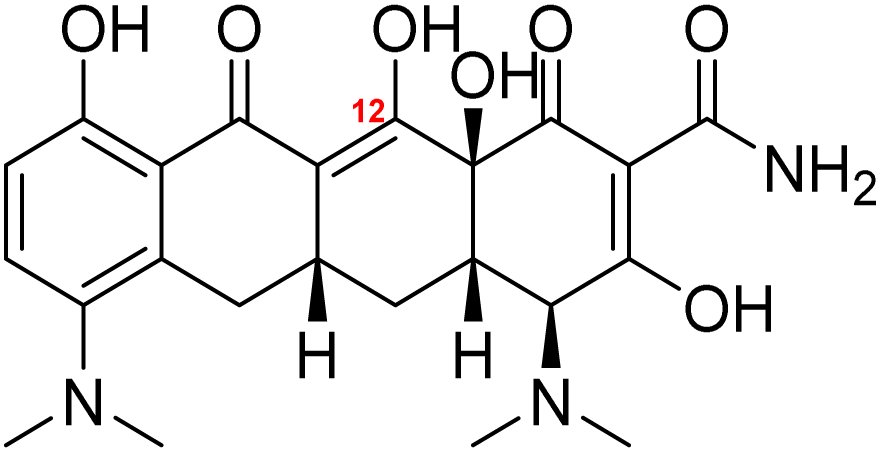	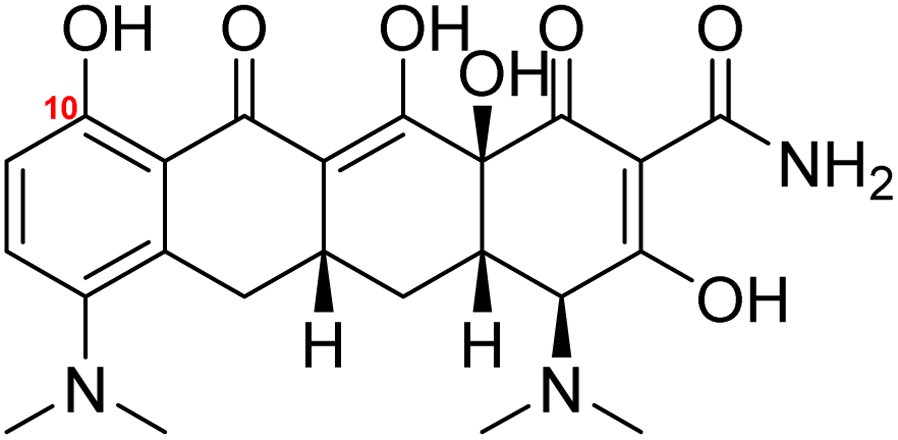	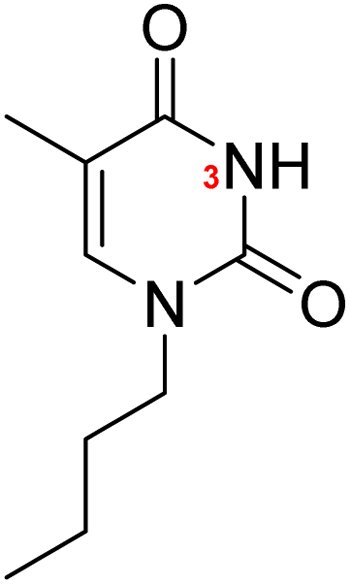
MC or 5 alone in 500 µL CDCl_3_ + 50 µL DMF-*d*_7_	14.56 ppm	11.43 ppm	8.89 ppm
MC + 5 in 500 µL CDCl_3_ + 50 µL DMF-*d*_7_	14.49 ppm	11.61 ppm	10.00 ppm
MC + 5 in 450 µL CDCl_3_ + 100 µL DMF-*d*_7_	14.51 ppm	11.59 ppm	9.89 ppm

To verify the H-bond formation, compound 5 and minocycline were each dissolved separately in a mixture of 0.25 mL CDCl_3_ and 25 µL DMF-*d*_7_. The two solutions were then combined, and the ^1^H NMR spectrum of the resulting mixture was recorded (Fig. S3). Upon mixing, a distinct deshielding of the N3–H proton of the thymine (Δ*δ* = 1.11 ppm) and the C10–OH proton of minocycline (Δ*δ* = 0.18 ppm) was observed (Table 1, line 4). A downfield shift of the N3–H proton has previously been reported for the uracil base when complexed, compared to its free form.^44^ In contrast, the hydroxyl proton in C12 of MC showed no significant shift (Δ*δ* = 0.07 ppm), suggesting that it was not involved in hydrogen bonding interactions (Fig. S4).

To assess the stability of these H-bonds, a ^1^H NMR spectrum was recorded upon adding a higher concentration of DMF-*d*_7_ (100 µL), competing with the intermolecular H-bond formed between compound 5 and MC (Table 1, line 5). If decomplexation occurs, changes in the chemical environment of the C10–OH and N3–H protons would be expected. In particular, compared to the spectrum of the complex (MC + 5 in 500 µL CDCl_3_ + 50 µL DMF-*d*_7_, Table 1, line 4), the proton signals of C10–OH and N3–H were expected to shift toward the positions of the individual unbound molecules. Upon addition of a higher concentration of DMF-*d*_7_, no significant shifts were observed for either the C10–OH or N3–H protons, indicating that the hydrogen bonds formed between minocycline and the thymine moiety of compound 5 remained strong and stable, even in the presence of a competitive solvent. Interestingly, although the C12–OH proton did not shift upon the addition of compound 5, it also remained unchanged upon increasing the concentration of DMF-*d*_7_. This suggests that this proton may already be engaged in interactions with the initial amount of DMF, potentially preventing hydrogen bond formation with the C4=O carbonyl group of thymine. These observations lead to the conclusion that the complexation of MC with compound 5 involves the formation of two strong and stable hydrogen bonds, rather than the three initially anticipated (Scheme S4).

The ability of CNDs and CND-L1 to complex MC and the ratio between the two components were evaluated by mixing the antibiotic (100 µg mL^−1^) with each type of CNDs (300 µg mL^−1^) for 24 h. For CND-L1, after complexation, the sample was centrifuged, and the supernatant was analyzed using UV-vis spectroscopy. The absorbance at 342 nm was compared with that of minocycline at 100 µg mL^−1^ (Fig. 2f). From the difference between the two absorption signals, it was determined that 56% of MC remained in solution, meaning that 44%, corresponding to 44 µg of drug, was complexed *via* hydrogen bonding to the thymine moiety of CND-L1. In the case of pristine CNDs, no sedimentation was observed after centrifugation up to 50 000 g, either for the CNDs alone or for the CNDs/MC complex. This is consistent with their small size (5–10 nm) and strongly negative zeta potential, which ensure high colloidal stability, whereas CND-L1 could be sedimented owing to its aggregation. The amount of free MC, in the case of pristine CNDs, could therefore not be determined from a supernatant, and HPLC was used instead. Free MC elutes as a single peak at 6.47 min, whose area was calibrated against MC standards (0.05–1 mg mL^−1^, Fig. S5). The CNDs/MC dispersions obtained after adding increasing amounts of MC (100 and 300 µg per 300 µg of CNDs) were injected directly, and the free MC remaining in solution was quantified from the area of this peak. No peak corresponding to free MC was detected for either amount (Fig. S6), indicating that the antibiotic was adsorbed onto the CNDs up to a 1 : 1 mass ratio. To allow a direct comparison of the antibacterial activities of CNDs and CND-L1, both nanomaterials were loaded with the same amount of MC. Since CND-L1 retained at most 44 µg of MC per 300 µg, whereas pristine CNDs could adsorb up to 300 µg, the loading capacity of CND-L1 was the limiting factor. The MC : CND ratio was therefore set to 40 µg per 300 µg of carbon nanodots (13.5% w/w), slightly below the maximum capacity of CND-L1, to ensure that all the drug is bound for both materials and that the two formulations contain identical amounts of MC and carbon nanodots. The amount of minocycline complexed was quite low and direct quantitative comparison of the minocycline loading with previously reported graphene oxide-based systems could not be performed as the exact loading amounts were not specified.^45,46^

### 3.3 Antimicrobial effect of carbon nanodots

Following the successful complexation of minocycline with CNDs and CND-L1, we evaluated the antibacterial efficacy of these formulations against *A.a.* The complexes used for the biological experiments were prepared at the loading ratio described in Section 3.2 (13.5%, w/w). CND-L1 is obtained as a dispersion at 240 µg mL^−1^, and the CNDs, obtained at 700 µg mL^−1^, were diluted to the same concentration so that the two materials could be directly compared. After complexation, CND-L1/MC was centrifuged, washed and redispersed in the same volume, so that the CND concentration of the stock remains 240 µg mL^−1^ and the corresponding MC concentration is 32.4 µg mL^−1^. CNDs/MC was used without purification, since HPLC showed no residual free MC. The stock and final assay concentrations, including the two dilution steps, are reported in Section 2.9. This concentration of MC can be considered subtherapeutic, as it is approximately 13-fold lower than the gingival crevicular fluid concentration (∼340 µg mL^−1^) achieved by commercially available minocycline microspheres, and free MC showed no antibacterial activity at this concentration (Fig. S7, SI).^40^

Colony counting assays revealed striking differences among the different treatment groups (Fig. 5a). Neither MC alone (96% survival), CNDs alone (100% survival), nor CND-L1 alone (92% survival) demonstrated significant antibacterial activity compared to the PBS control (100% survival, *P* > 0.05 for all comparisons) (Fig. 5b). The CNDs/MC complex also showed minimal antibacterial effect (99% survival, *P* = 0.9997 *vs.* control), indicating that a simple complexation of MC with pristine CNDs did not confer antibacterial activity at subtherapeutic concentrations of minocycline. The CND-L1/MC complex reduced bacterial survival to 75% of the control (*P* = 0.0003, Fig. 5b).

**Fig. 5:**
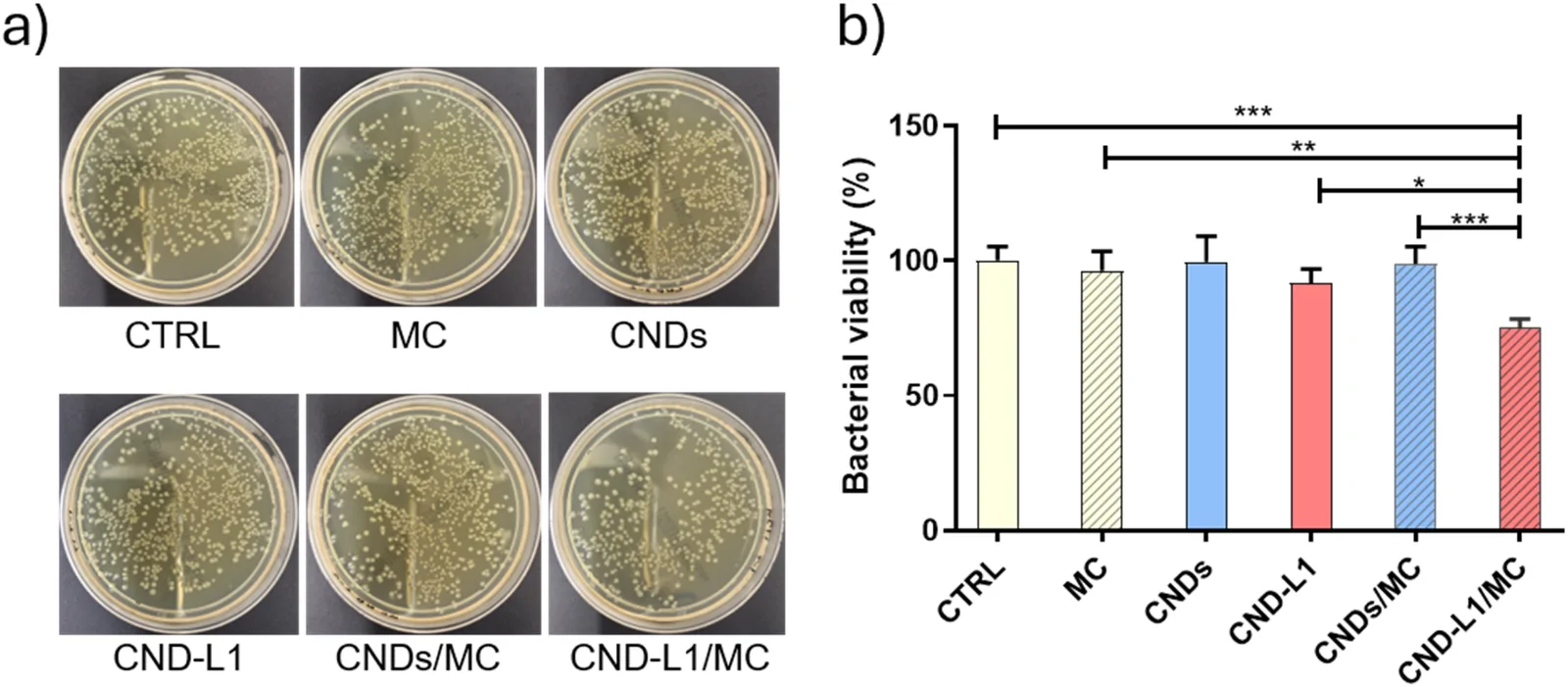
Colony-forming unit (CFU) counts of *A.a.* after the different treatments (CTRL, MC, CNDs, CND-L1, CNDs/MC, CND-L1/MC). (a) Representative images of colony counting assay for each treatment group. (b) Bacterial viability (%) relative to the PBS control. Data are expressed as mean ± standard deviation from four independent experiments (*n* = 4 plates per experiment). Statistical analysis was performed using one-way ANOVA, followed by Tukey's *post hoc* test. *P < 0.05, **P < 0.01, ***P < 0.001.

Dose-response experiments confirmed that the concentration of MC used in this study represented a subinhibitory concentration of MC. MC alone at 25.6 µg mL^−1^ showed no significant antibacterial effect (100% survival rate compared to control), whereas 10-fold (256 µg mL^−1^) and 100-fold (2560 µg mL^−1^) concentrations resulted in effective bacterial killing (2% and 0% survival, respectively; Fig. S7b). This confirmed that the CND-L1/MC complex is active at a concentration at which MC alone is ineffective. Since the CNDs showed no antibacterial activity of their own under these conditions, the difference between CNDs/MC and CND-L1/MC therefore arises from the way MC is bound in each complex.

This effect, which was observed only when MC was complexed with L1-modified CNDs, suggests that L1 ligand modification plays a critical role in enabling antibacterial activity at subtherapeutic concentrations of minocycline.

### 3.4 Mechanisms of action of carbon nanodots

The above results show that complexation of minocycline with CND-L1 increases the antibacterial effect of the antibiotic at a concentration at which the free drug is inactive. This motivated further investigation into the underlying antibacterial mechanism of CND-L1/MC.

Antibacterial properties of CNDs are widely reported in the literature, and a few principal mechanisms have been proposed to explain this type of activity, which we summarize below as possible contributions rather than as effects demonstrated in the present system.^47–50^ One mechanism involves the interactions of CNDs with the bacterial membrane inducing structural damages or affecting the functionality and transportation of cellular compounds resulting in bacterial death and inability to proliferate.^48,51–53^ Another mechanism relies on the internalization of the CNDs by the bacteria mainly by diffusion, followed by the generation of reactive oxygen species (ROS). Normally, ROS in bacterial cells are regulated by their oxidoreductase system. When the level of ROS increases, it can cause some irreversible oxidative damages to the bacterial cells such as morphological changes of the cell membrane, cytoplasm leakage and DNA impairment.

#### 3.4.1 Cellular localization and bacterial association

To elucidate the mechanism underlying the enhanced antibacterial activity of CND-L1/MC, we evaluated the interaction between the different CNDs and the bacterial cells, both as single components and in complex with MC, using confocal laser scanning microscopy (CLSM) and quantitative flow cytometry (FACS). Viable *A.a.* cells were incubated with CNDs, CND-L1, CNDs/MC, or CND-L1/MC, fixed after exposure, and then stained with Hoechst 33342 to label the bacterial nucleoids. The intrinsic red autofluorescence of CNDs (excitation 488 nm, emission 600–720 nm) enabled their direct visualization and quantification without additional labeling.

CLSM images showed red fluorescence signals from CNDs and CND-L1 colocalized with the blue Hoechst signal of *A.a*. nucleoids in both single-component and MC-complexed conditions (Fig. 6a–d), indicating that the carbon nanodots and their MC complexes physically associate with the bacterial cells. Visually, the red signal from CND-L1/MC appeared stronger than that from CNDs/MC under the same exposure conditions, suggesting a difference in the extent of bacterial association after MC loading. A subset of bright-field-visible cells lacked a corresponding fluorescence signal, consistent with these cells lying outside the single confocal optical plane used for imaging (see Methods) rather than reflecting an absence of dye uptake or particle binding.

**Fig. 6:**
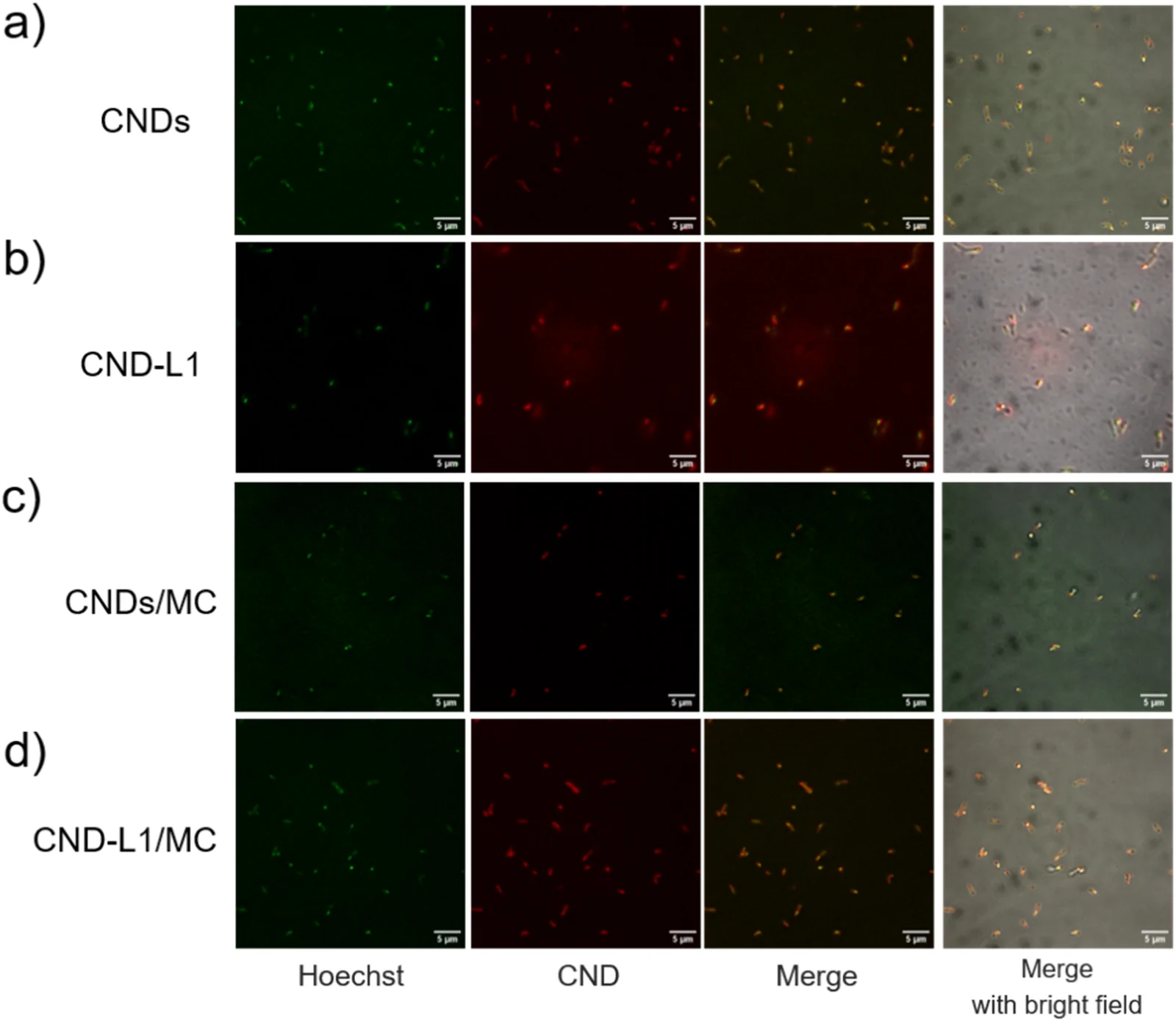
Interaction of CNDs and their MC complexes with *A.a*. (a–d) Confocal laser scanning microscopy images of *A.a.* incubated with (a) CNDs, (b) CND-L1, (c) CNDs/MC, or (d) CND-L1/MC. Blue: Hoechst 33342 (excitation 403 nm, emission 420–480 nm); red: CND or CND-L1 autofluorescence (excitation 489 nm, emission 600–720 nm). Carbon nanodots were used at 218 µg mL^−1^ for both CNDs and CND-L1, and the complexes contained 218 µg mL^−1^ of carbon nanodots with 29 µg mL^−1^ of MC. Bacteria were exposed while viable and fixed only after exposure. Bright-field-visible cells lacking fluorescence signal reflect their position outside the single imaged focal plane (no *z*-stack), not an absence of labeling. Scale bars: 5 µm.

To quantify this observation, FACS analysis was performed. The bacterial cells were gated as Hoechst-positive events (Fig. S8a) and the CND-derived fluorescence intensity was measured in the PE channel (PE-H), capturing the CND red emission. Within the Hoechst-positive bacterial gate, CND-L1/MC produced a PE-H median fluorescence intensity approximately 4-fold higher than CNDs/MC (Fig. 7). A no-bacteria control (CND-L1/MC suspended in PBS without *A.a.*) showed approximately 1% of events falling into the bacterial gate (Fig. S8b), confirming that the measured PE-H signal in the experimental samples reflects genuine complex–bacteria association rather than free particles in suspension.

**Fig. 7:**
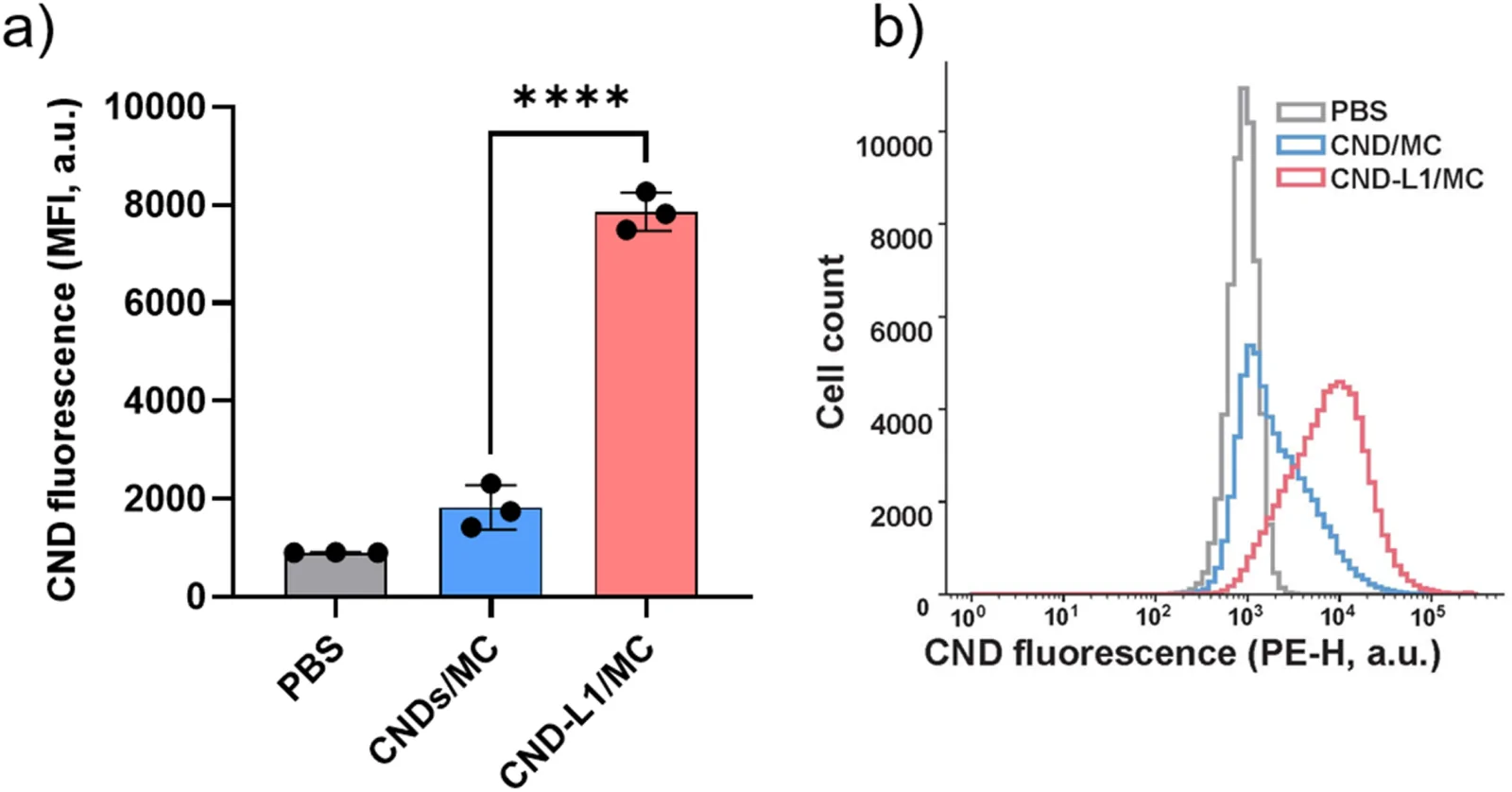
(a) Flow-cytometric quantification of CNDs fluorescence associated with the bacteria, expressed as the median fluorescence intensity (MFI) of the CND signal detected in the phycoerythrin channel (PE-H) within the Hoechst-positive bacterial gate (Pacific Blue-H > 100). Bars represent mean ± SD of three technical replicates; ****p < 0.0001 (Welch's *t*-test). (b) Histograms of CND fluorescence in the PE channel for events within the Hoechst-positive bacterial gate. CND-L1/MC produced a rightward shift relative to CNDs/MC. One representative replicate of three is shown. The specificity of the bacterial gate, including the no-bacteria control, is presented in Fig. S8.

Taken together, the CLSM and FACS results indicate that the L1 modification of CNDs largely preserves the complex–bacteria association under MC loading. The ∼4-fold higher association of CND-L1/MC with *A.a.* compared with CNDs/MC is consistent with, and may contribute to, the enhanced antibacterial activity observed for CND-L1/MC at subtherapeutic MC concentrations. We do not claim that bacterial association alone determines the antibacterial outcome; however, under our experimental conditions (MC final concentration 25.6 µg mL^−1^), the difference in complex–bacteria association quantitatively parallels the difference in antibacterial activity observed in Fig. 5.

#### 3.4.2 ROS generation by carbon nanodots

ROS levels in bacterial cells are regulated by redox enzyme systems to maintain a dynamic equilibrium. Elevated intracellular ROS are considered as a primary mechanism responsible for bacterial cell death. The ability of carbon dots to generate ROS has been widely reported, using mainly irradiation, conferring to the carbon dots an enzyme-like activity.^53–56^ Moreover, this ability has been exploited for antibacterial applications.^53,55,57,58^

Considering that CNDs and their MC complexes associate with *A.a.* cells, we next investigated whether the antibacterial activity is associated with ROS generation. ROS generation was evaluated using dihydrorhodamine 123 (DHR123) assay under dark conditions (no irradiation and protection from light) and upon irradiation with 808 nm or 660 nm lasers. Under dark conditions neither CNDs (Fig. S9) nor CND-L1 (Fig. S12) produced ROS indicating that both carbon dots are inactive in the absence of light. Upon irradiation with laser 808 nm, CND-L1 exhibited a significant increase in fluorescence emission at 534 nm (Fig. S14) indicating ROS generation, whereas CNDs remained inactive (Fig. S11). ROS production by CND-L1 was even more pronounced upon 660 nm irradiation (Fig. S13) confirming its efficient light-induced ROS generation, while CNDs continued to show no ROS production (Fig. S10). Two control experiments were conducted to confirm the role of the ligand L1 in ROS production. First, the free ligand L1, which was not covalently attached to CNDs, was tested and showed no ROS production under the present conditions (Fig. S15). Second, pristine CNDs were evaluated in the presence of the free ligand L1 in solution. Under these conditions, no fluorescence emission at 534 nm was detected, indicating the absence of ROS generation by CNDs (Fig. S16–S18). These results demonstrate that ROS production requires the covalent functionalization of ligand L1 onto CNDs. Under the conditions tested here, ROS generation upon irradiation was observed for CND-L1 but not for the unmodified CNDs, indicating that this behavior is associated with the presence of the thymine ligand. Light-induced ROS generation by carbon dots has been reported in the literature.^53–55^ Additionally, the aggregation of nanomaterials was shown to promote in some cases ROS generation.^59,60^ Accordingly, the enhanced ROS level observed for CND-L1 may arise from the tendency of the carbon dots to interact *via* hydrogen bonding mediated by the thymine moiety and subsequently forming aggregated species. Although ROS production after irradiation could potentially be exploited for antibacterial applications, no light exposure was applied during the antibacterial assays in this study. Therefore, ROS generation is unlikely to be the primary mechanism responsible for the antibacterial activity of CND-L1/MC under the experimental conditions used.

#### 3.4.3 Considerations on additional mechanisms

Beyond the bacterial association documented above, we also considered whether ROS generation or direct DNA interaction could account for the difference in antibacterial activity between CND-L1/MC and CNDs/MC. As described in Section 3.4.2, ROS generation by CNDs and CND-L1 required laser irradiation and was therefore not relevant under the present non-irradiated assay conditions. Direct DNA interaction was assessed by agarose gel electrophoresis and circular dichroism (Fig. S19, SI). Neither of these processes followed the pattern of antibacterial activity observed for CND-L1/MC at subtherapeutic MC doses. Taken together, these observations place the origin of the difference in the complex itself rather than in an intrinsic activity of the carbon nanodots. The hydrogen bonds formed between the thymine unit and MC are what hold the antibiotic on the CND-L1 surface, and the resulting complex associates approximately four times more with *A.a.* than CNDs/MC, bringing a larger amount of MC into contact with the bacteria. The binding mode is also expected to govern how MC is subsequently released once the complex has reached the bacteria. A mixture of free MC and free L1, without carbon nanodots, was not included in the antibacterial assays. Such a mixture is a different system, without a carrier, and would not inform the comparison between CNDs/MC and CND-L1/MC on which this study rests.

### 3.5 Cytotoxicity of carbon nanodots on osteoblast-like cells

Any locally applied antimicrobial agent has to act selectively on bacteria without damaging the surrounding host cells, and the relevant cell type depends on where the agent is used. At the periodontal and peri-implant sites where local MC delivery is commonly employed, bone constitutes a major component of the surrounding tissue, and osteoblasts are therefore the cell type whose response is primarily relevant. We evaluated the cytotoxicity of all CND formulations toward MC3T3-E1 murine osteoblast-like cells over 48 h using the WST-1 assay (Fig. 8). The metabolic activity of all groups, including MC (0.66 µg mL^−1^), CNDs (5.0 µg mL^−1^), CND-L1 (5.0 µg mL^−1^), CNDs/MC and CND-L1/MC, increased over the 48 h period and remained above 70% of the untreated control at every time point, with no significant difference from the control at 4, 8, 24 or 48 h.

**Fig. 8:**
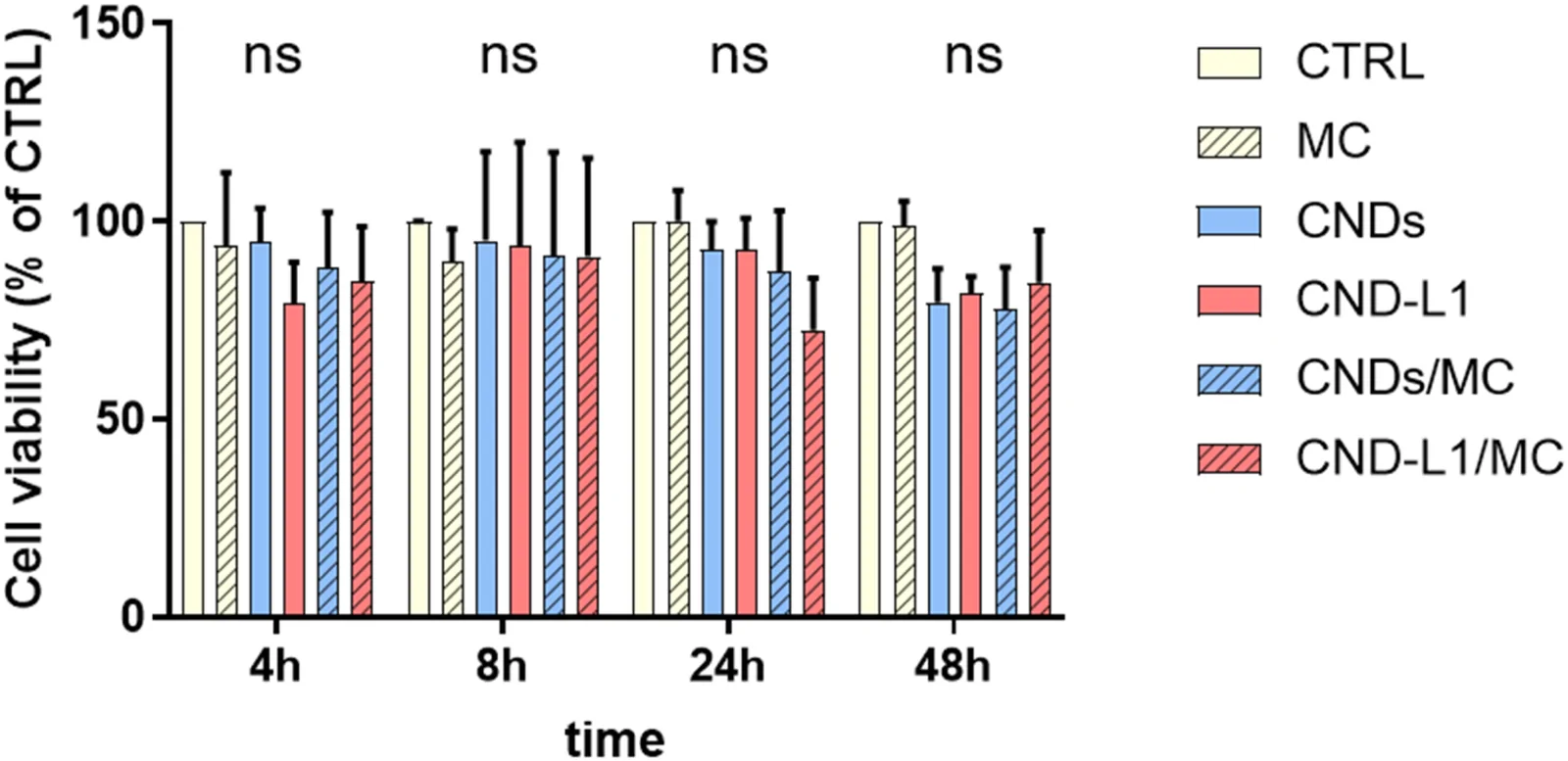
Metabolic activity of MC3T3-E1 osteoblast-like cells exposed to PBS (CTRL), MC, CNDs, CND-L1, CNDs/MC, or CND-L1/MC for 4, 8, 24, and 48 h, measured by WST-1 assay. Data are expressed as cell viability (%) relative to the CTRL group at each time point (mean ± SD of three independent experiments, each performed with five wells per group). Statistical analysis was performed using one-way ANOVA followed by Tukey's *post hoc* test; ns: not significant.

The absence of any reduction in metabolic activity is relevant to the intended application, since an antibacterial treatment applied close to an implant must not compromise the osseointegration on which implant stability depends.

Peri-implantitis represents a clinically significant example of this challenge. Dental implants are widely used for the replacement of missing teeth,^61^ but their long-term success is limited by peri-implantitis, which is observed in 11.7–13.4% of implants and 12.9–26.2% of patients within 5–10 years after placement.^62^ Current treatments, including mechanical debridement and local or systemic antibiotics, give variable outcomes,^63^ and the implant surface topography further restricts the delivery of antimicrobial agents.^64^ An agent that remains active at a reduced MC concentration while preserving osteoblast viability, as observed here for CND-L1/MC, addresses both sides of this problem.

The present study establishes the efficacy of CND-L1/MC in a defined single-species setting. Extending the evaluation to biofilm and multi-species models, and subsequently to *in vivo* systems, will determine whether the effect observed here translates to the more complex environment of an infected site.

## 4 Conclusions

In this study, we synthesized and characterized minocycline-loaded carbon nanodots (CNDs and CND-L1) and examined how the mode of drug binding affects MC loading and retention, the association of the loaded carrier with bacterial cells, and antibacterial activity. Only the CND-L1/MC complex reduced bacterial survival at subtherapeutic concentrations of minocycline, indicating that the ligand influences the behavior of the complex beyond drug loading alone. Quantitative flow cytometry analysis revealed that CND-L1/MC associates with *A.a.* cells approximately 4-fold more efficiently than CNDs/MC.

Overall, these findings show that introducing a hydrogen-bonding ligand onto carbon nanodots modulates not only drug loading but also the interaction of the resulting complex with bacterial cells.

## Author contributions

Conceptualization: Y. T., A. Y., A. B., E. H. Methodology: O. S., Y. T., E. H. Investigation: O. S., Y. T., J. Q., Y. M. Formal analysis: O. S., Y. T., J. Q., Y. M., E. H. Resources: Y. T., K. S., A. Y., A. B., E. H. Supervision: A. Y., A. B., E. H. Funding acquisition: Y. T., A. Y., A. B., E. H. Writing – original draft: O. S., Y. T., E. H. Writing – review & editing: all authors. All authors have read and approved the final version of the manuscript.

## Conflicts of interest

The authors declare no conflicts of interest to this work.

## Supplementary Material

NA-OLF-D6NA00753H-s001

## Data Availability

The data supporting the findings are available from the corresponding authors upon reasonable request. Supplementary information (SI) is available. See DOI: https://doi.org/10.1039/d6na00753h.
