# Supplementary material for: Red-emissive thymine-functionalized carbon nanodots potentiate minocycline antibacterial activity at subtherapeutic doses
Source: Nanoscale Adv. 2026 Nov 4. Online ahead of print. doi: 10.1039/d6na00753h (PMC13756210; doi:10.1039/d6na00753h)
Supplement: Supplementary file 1 [file NA-OLF-D6NA00753H-s001.pdf]

Supplementary Information (SI) for Nanoscale Advances.
This journal is © The Royal Society of Chemistry

Red-Emissive Thymine-Functionalized Carbon Nanodots Potentiate Minocycline Antibacterial Activity at Subtherapeutic Doses

Océane Seivert, Yuta Takano, Junyue Qu, Yukari Maeda, Kiwamu Sakaguchi, Atsuro Yokoyama, Alberto Bianco* and Eri Hirata,**

Electronic Supplementary Information

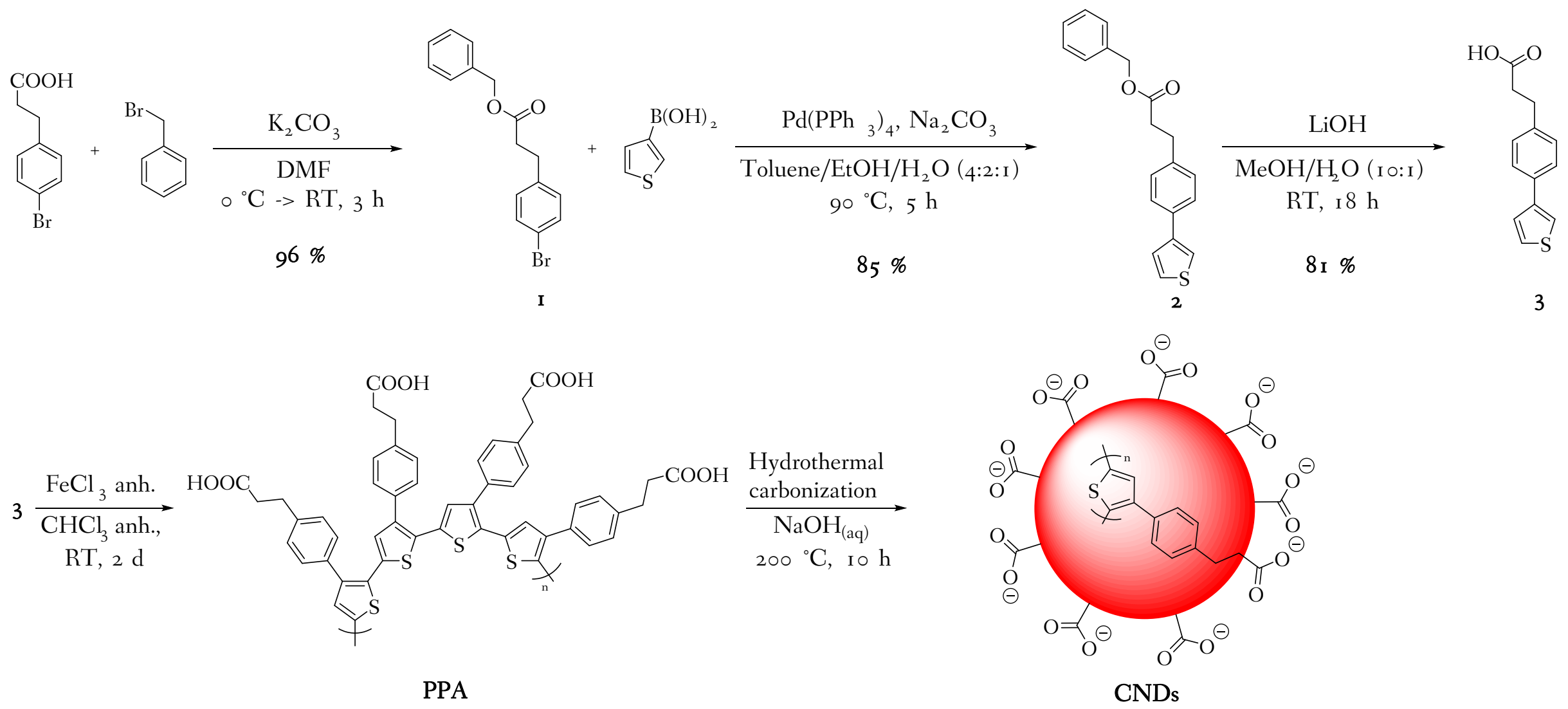

Scheme S1. Synthesis of CNDs.

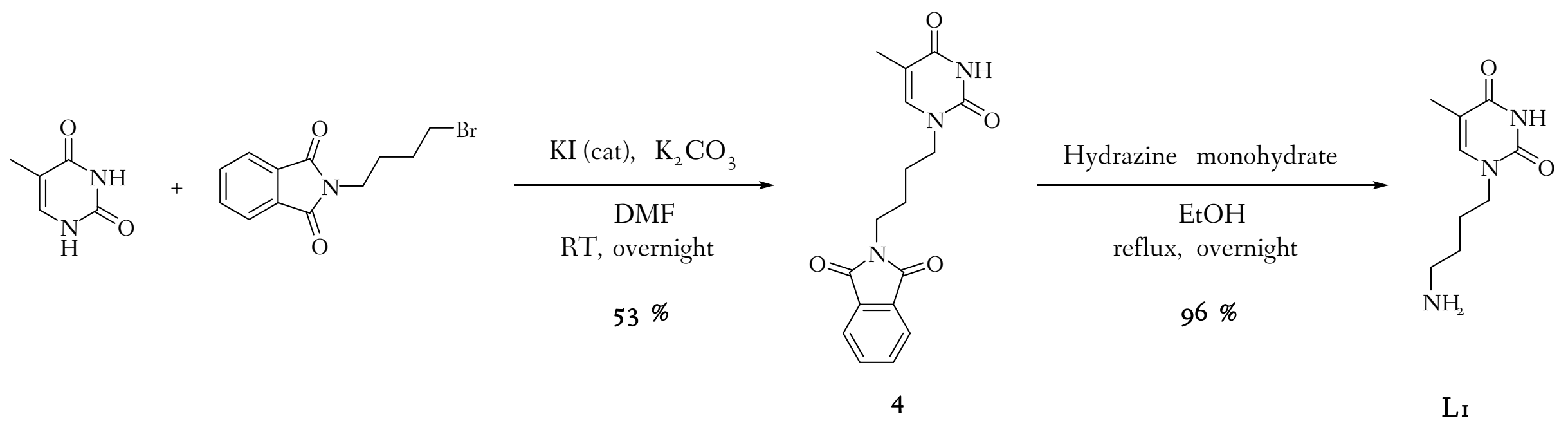

Scheme S2. Synthesis of ligand L1.

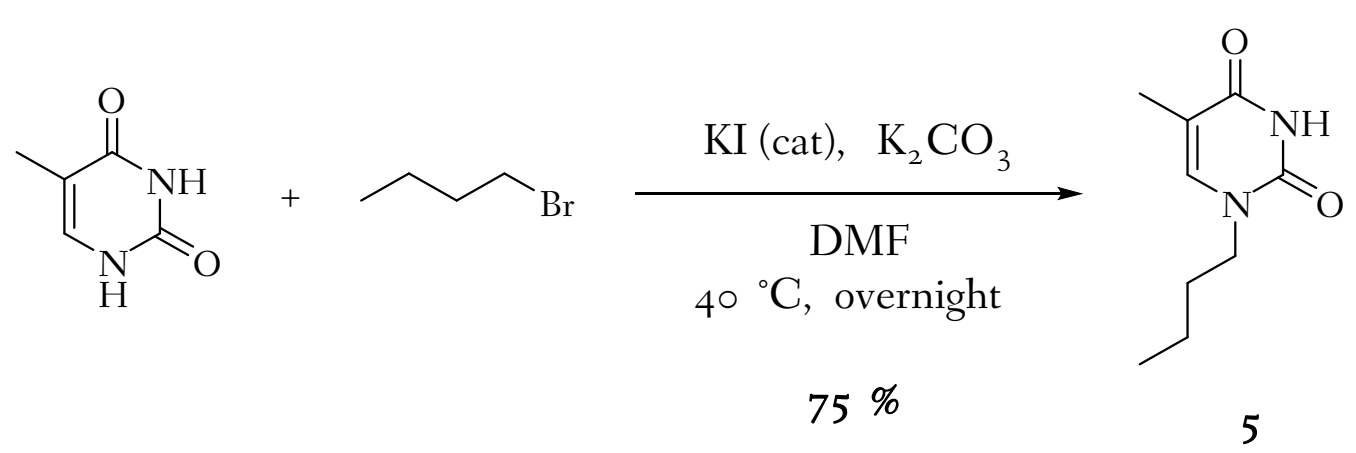

Scheme S3. Synthesis of compound **5**.

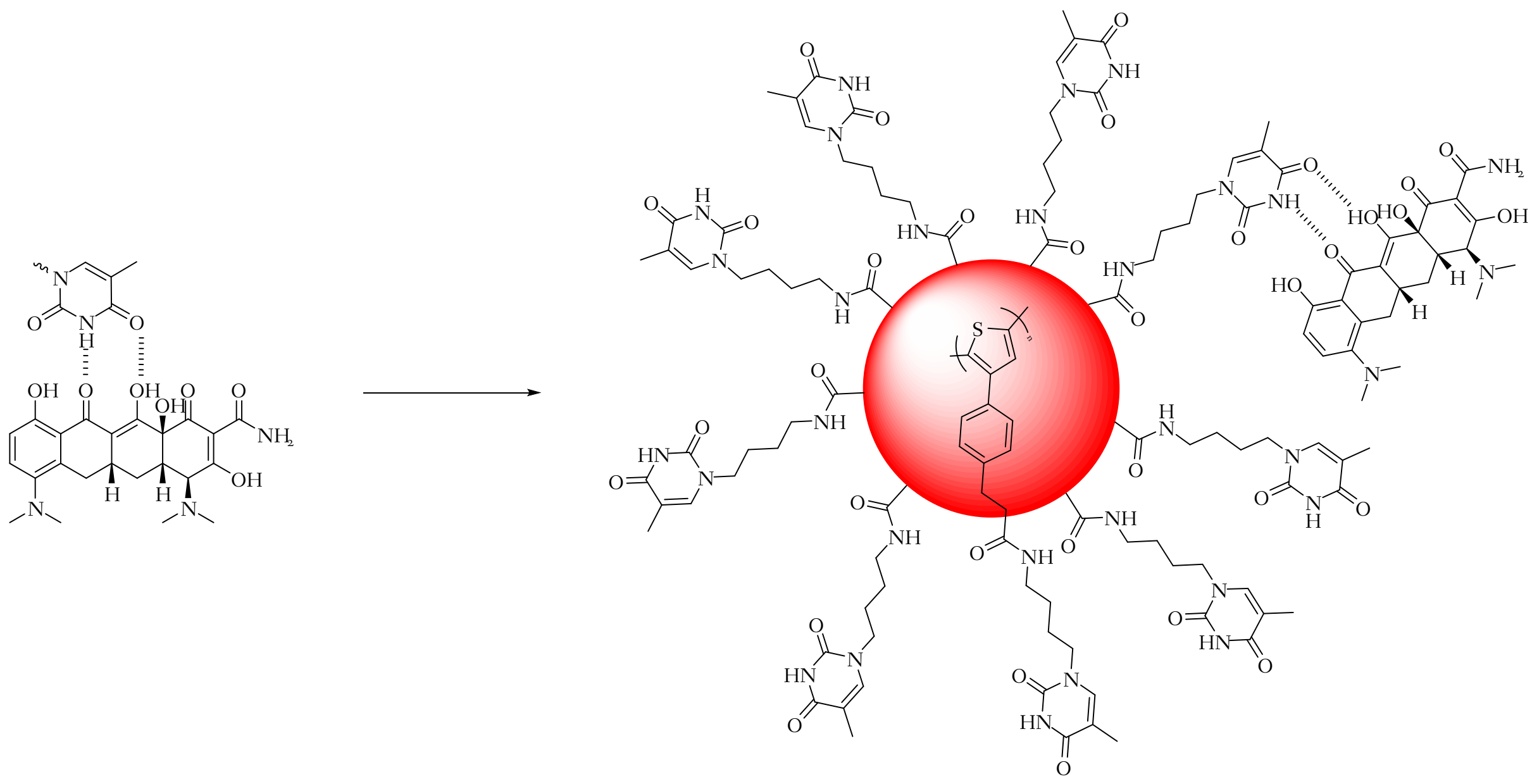

Scheme S4. Chemical representation of the hydrogen bond formation between the thymine moiety and the minocycline on CND-L1.

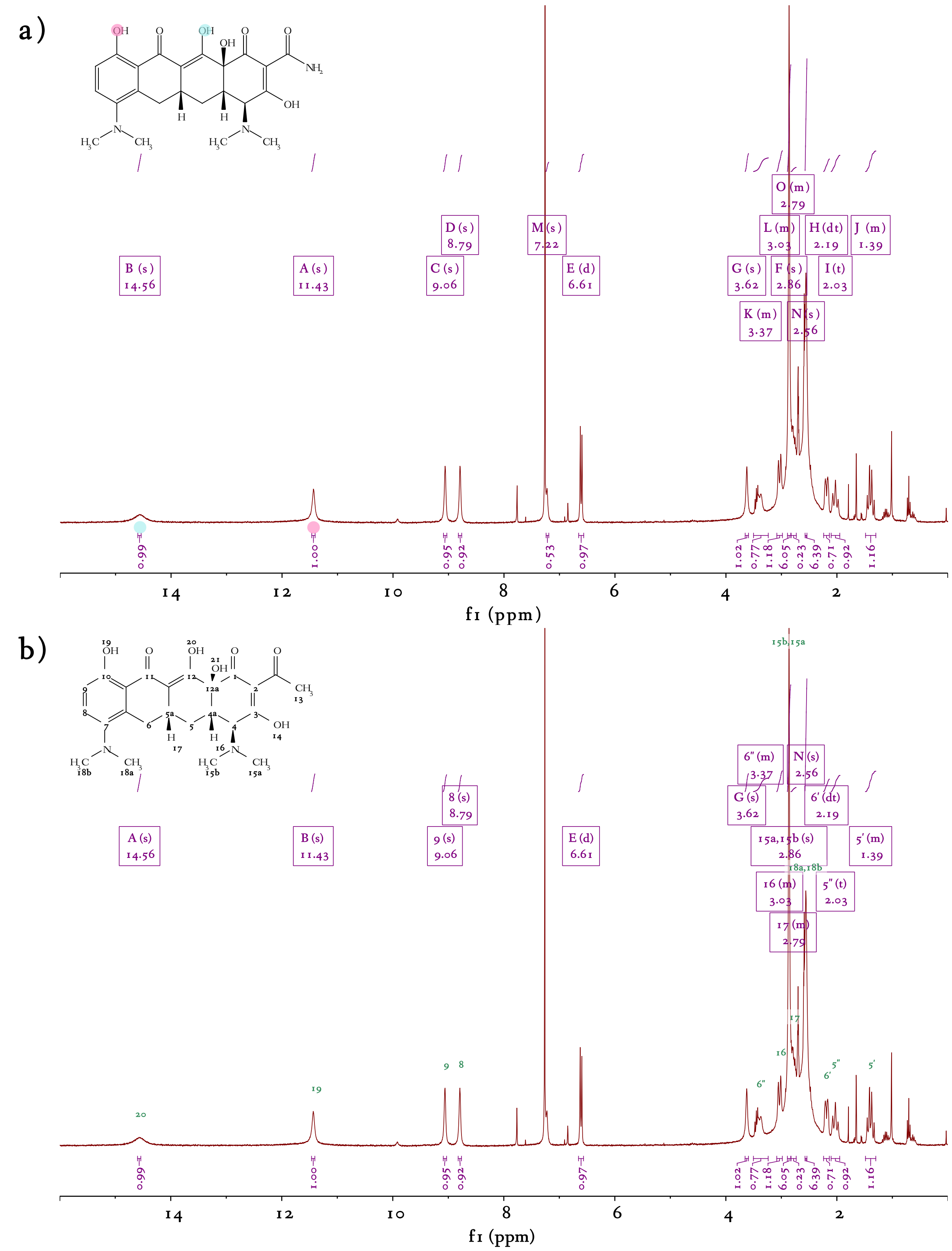

Figure S1. ^{1}H NMR spectrum of minocycline recorded in a mixture of $CDCl_3$ (500 μL) and DMF-d_7 (50 μL) showing a) color-coded assignment of protons involved in the intermolecular interactions with their corresponding shifts, and b) correlation between the numbered proton on the molecular structure and their chemical shift in the spectrum.

a)

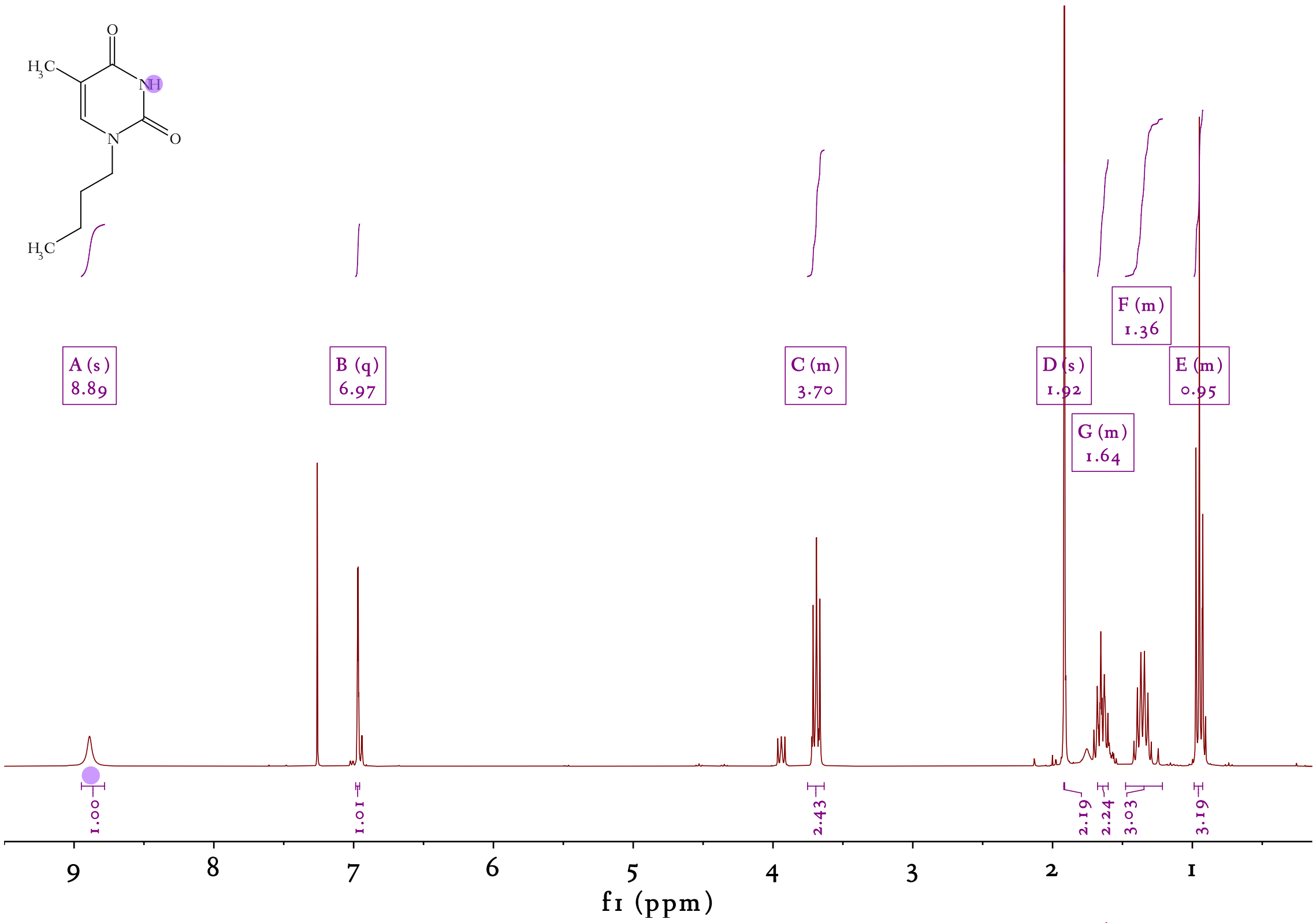

b)

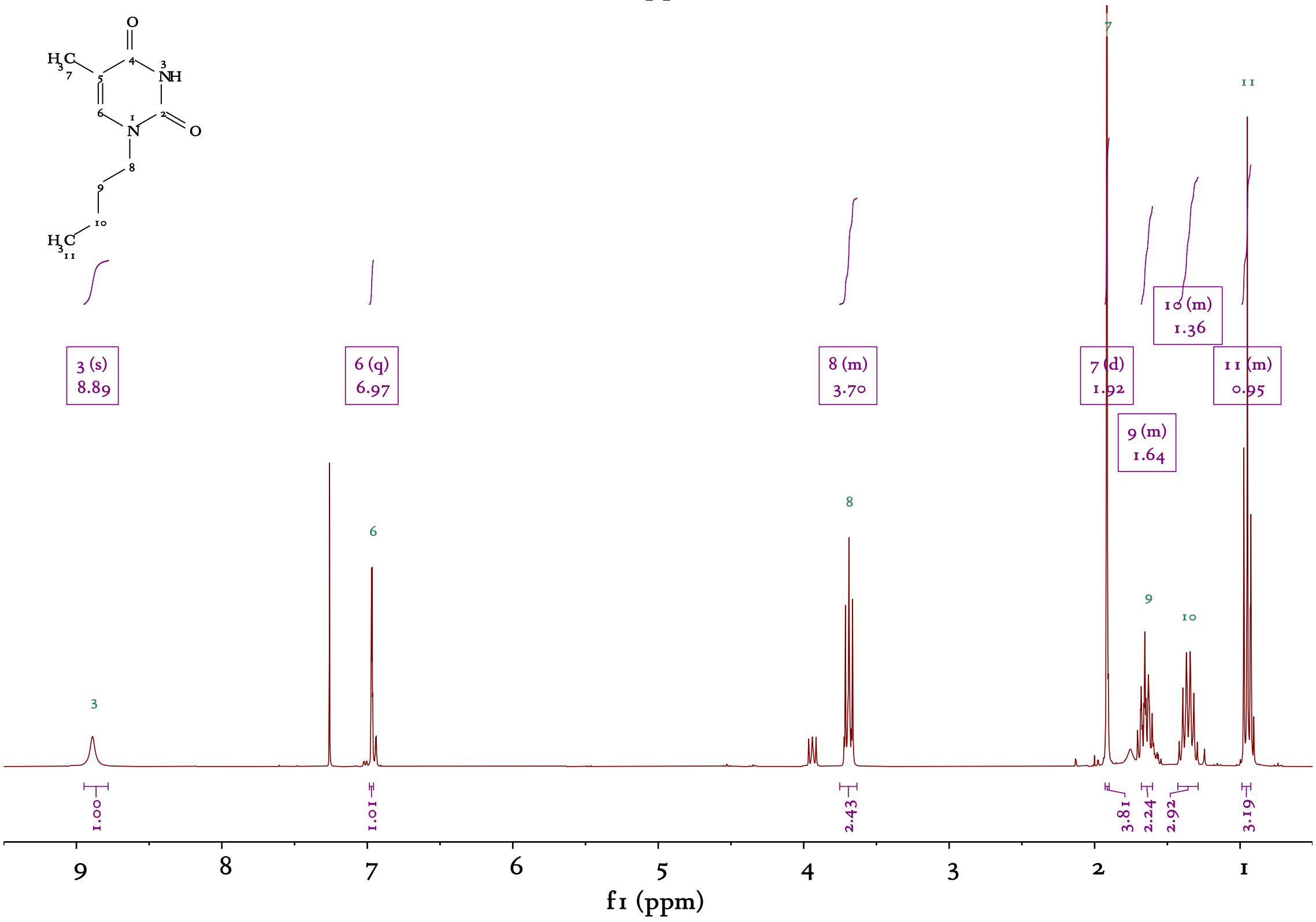

Figure S2. ¹H NMR spectrum of compound **5** recorded in a mixture of $CDCl_3$ (500 μL) and DMF-d_7 (50 μL) showing a) color-coded assignment of protons involved in the intermolecular interactions

with their corresponding shifts, and b) correlation between the numbered proton on the molecular structure and their chemical shift in the spectrum.

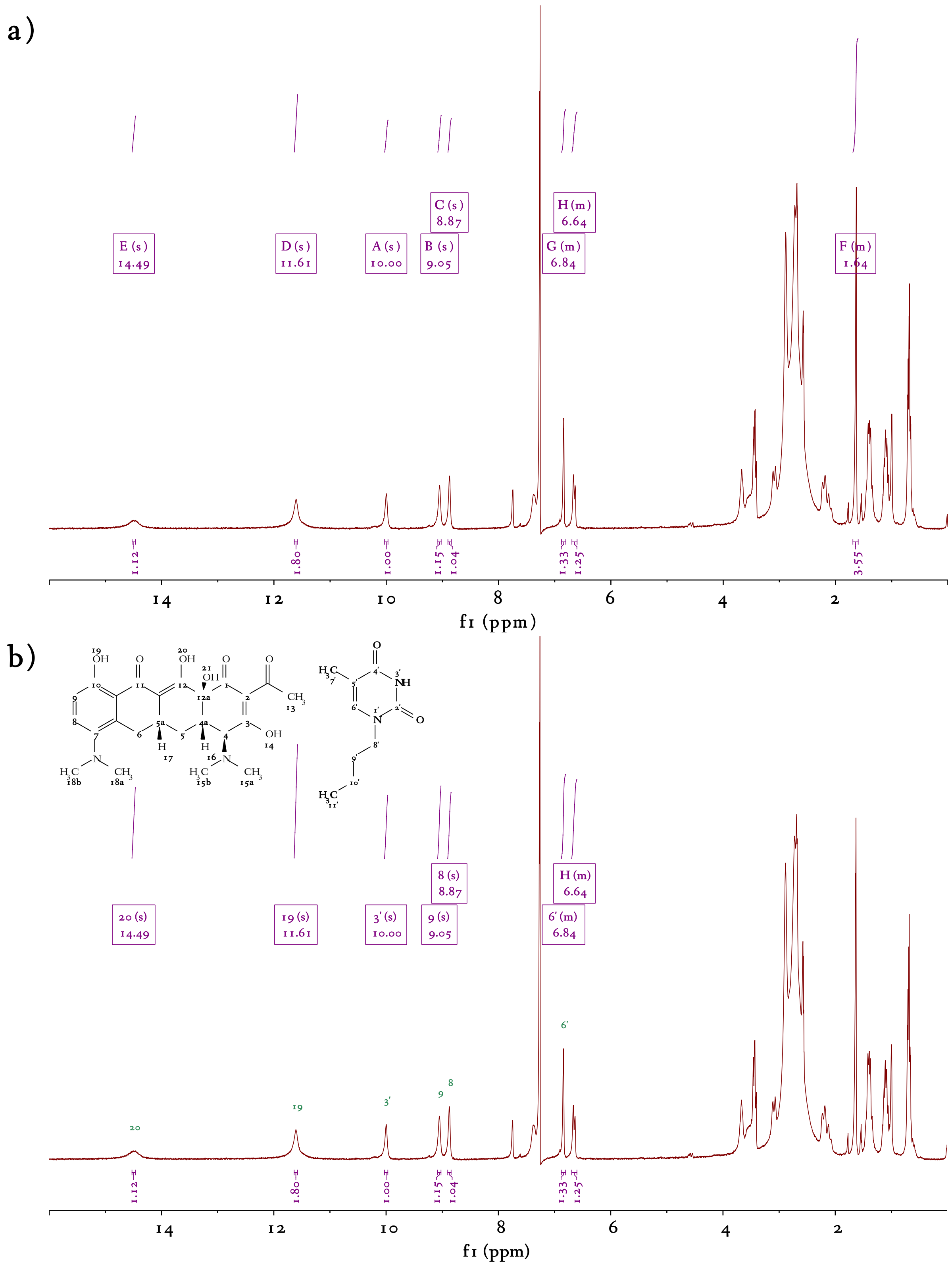

Figure S3. ^{1}H NMR spectrum of minocycline and compound **5** in a mixture of $CDCl_3$ (500 μL) and DMF-d_7 (50 μL) with a) the bare spectrum and b) correlation between the numbered proton on the molecular structure and their chemical shift in the spectrum.

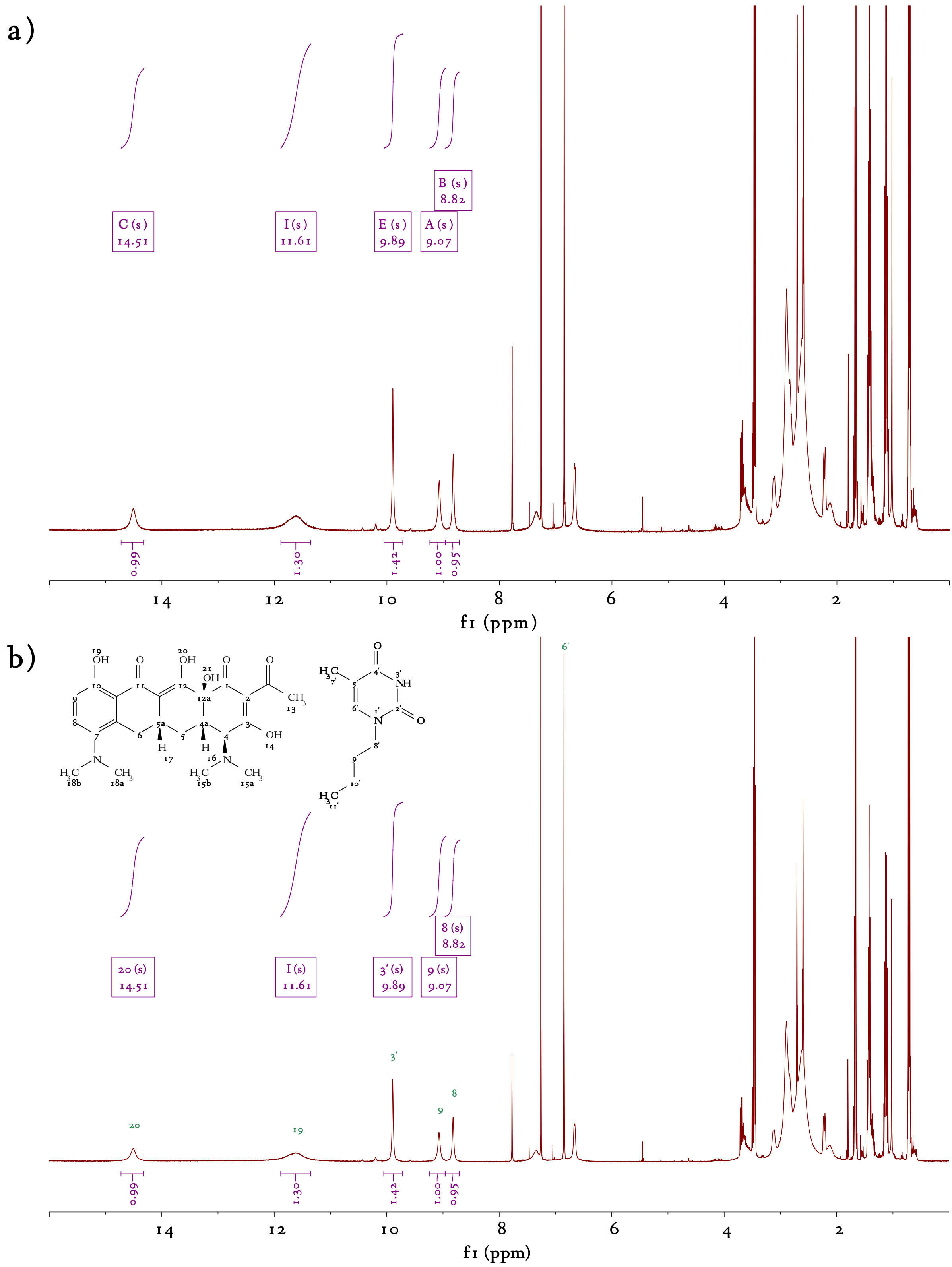

Figure S4. ^{1}H NMR spectrum of minocycline and compound **5** in a mixture of $CDCl_3$ (450 μL) and DMF-d_7 (100 μL) with a) the bare spectrum and b) correlation between the numbered proton on the molecular structure and their chemical shift in the spectrum.

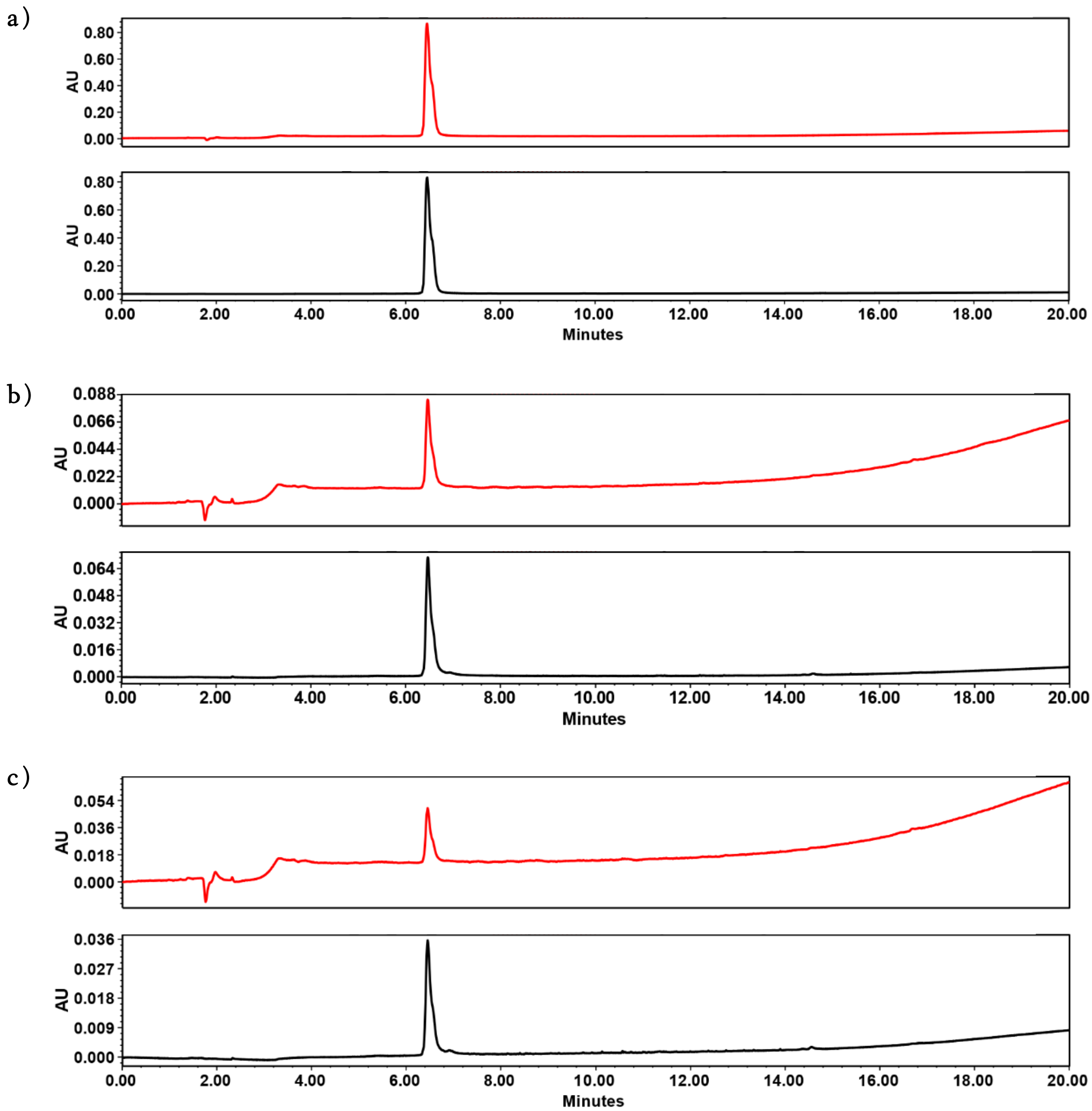

Figure S5. HPLC chromatograms of minocycline with a concentration of a) 1 mg mL^{-1}, b) 0.1 mg mL^{-1}, and c) 0.05 mg mL^{-1} showing a peak at 6.47 min. In red is displayed the spectrum monitored at 220 nm and in black is the spectrum monitored at 254 nm.

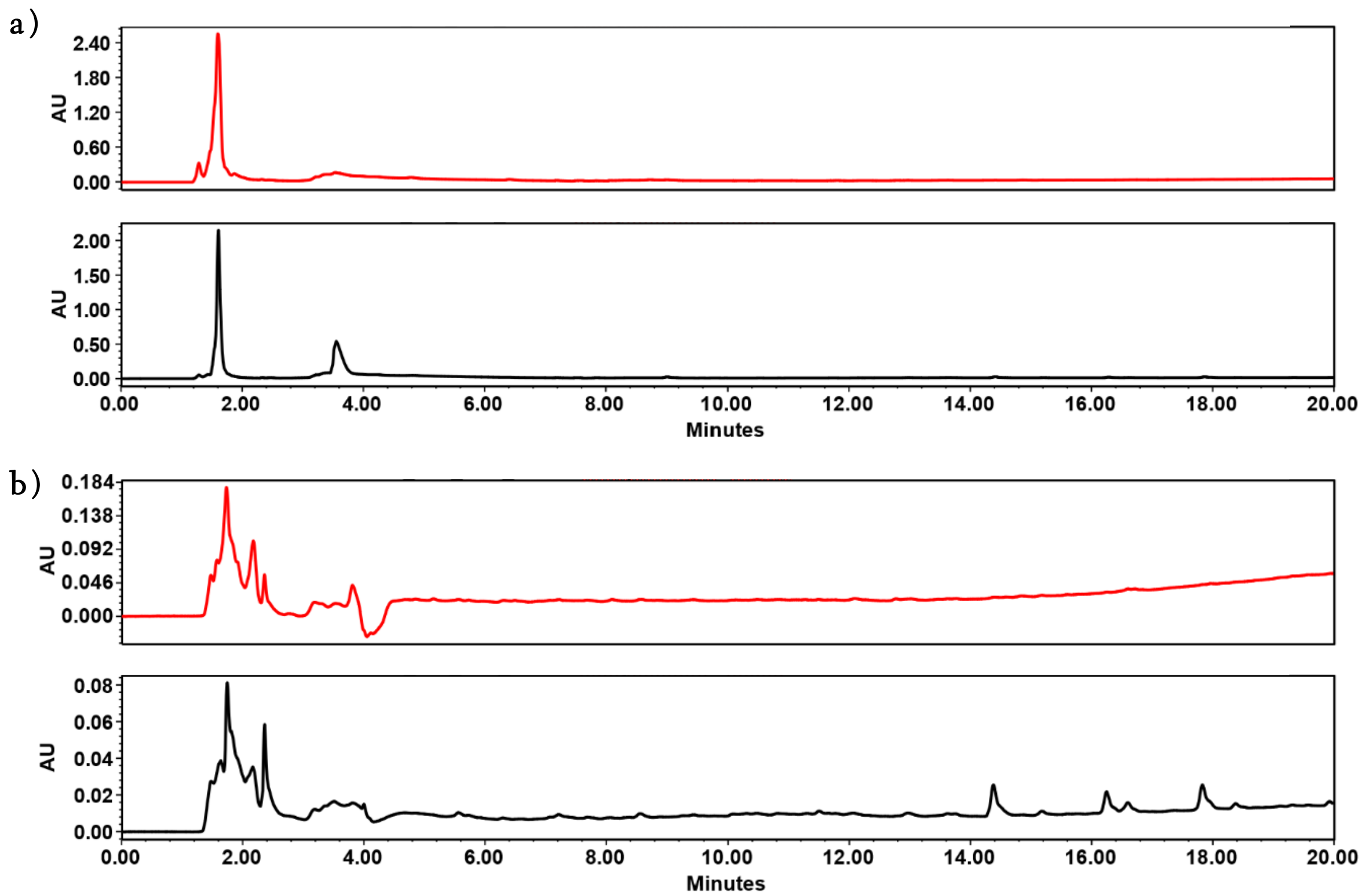

Figure S6. HPLC chromatograms of the solutions containing the conjugates CNDs/MC after adding (a) 100 μg of MC and (b) 300 μg of MC to a solution of CNDs in Milli-Q® water at a concentration of 300 μg mL^{-1}. In red is displayed the spectrum monitored at 220 nm and in black is the spectrum monitored at 254 nm.

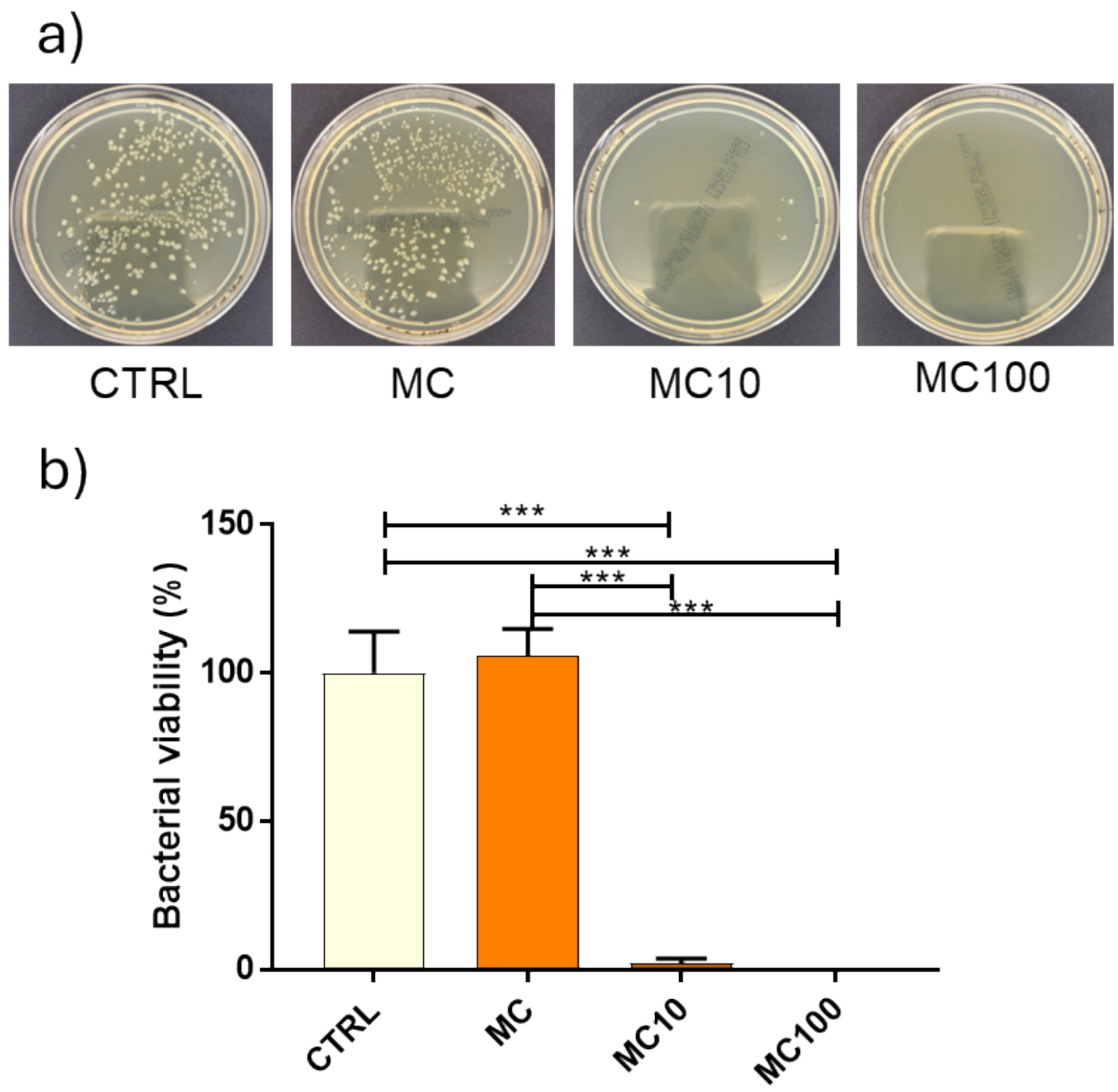

Figure S7. Dose-response experiment validating the minocycline concentration used in the antibacterial assays. (a) Representative images of agar plates from colony counting assays. (b) Quantification of bacterial viability expressed as percentage relative to PBS control (100%). Minocycline was tested at three concentrations: MC (25.6 μg mL^{-1}, 1×), MC10 (256 μg mL^{-1}, 10×), and MC100 (2560 μg mL^{-1}, 100×). Four independent experiments were conducted, with four agar plates per condition in each experiment. Values represent mean ± SD. Statistical significance was determined by one-way ANOVA with Tukey's multiple comparison test (***$P < 0.001$).

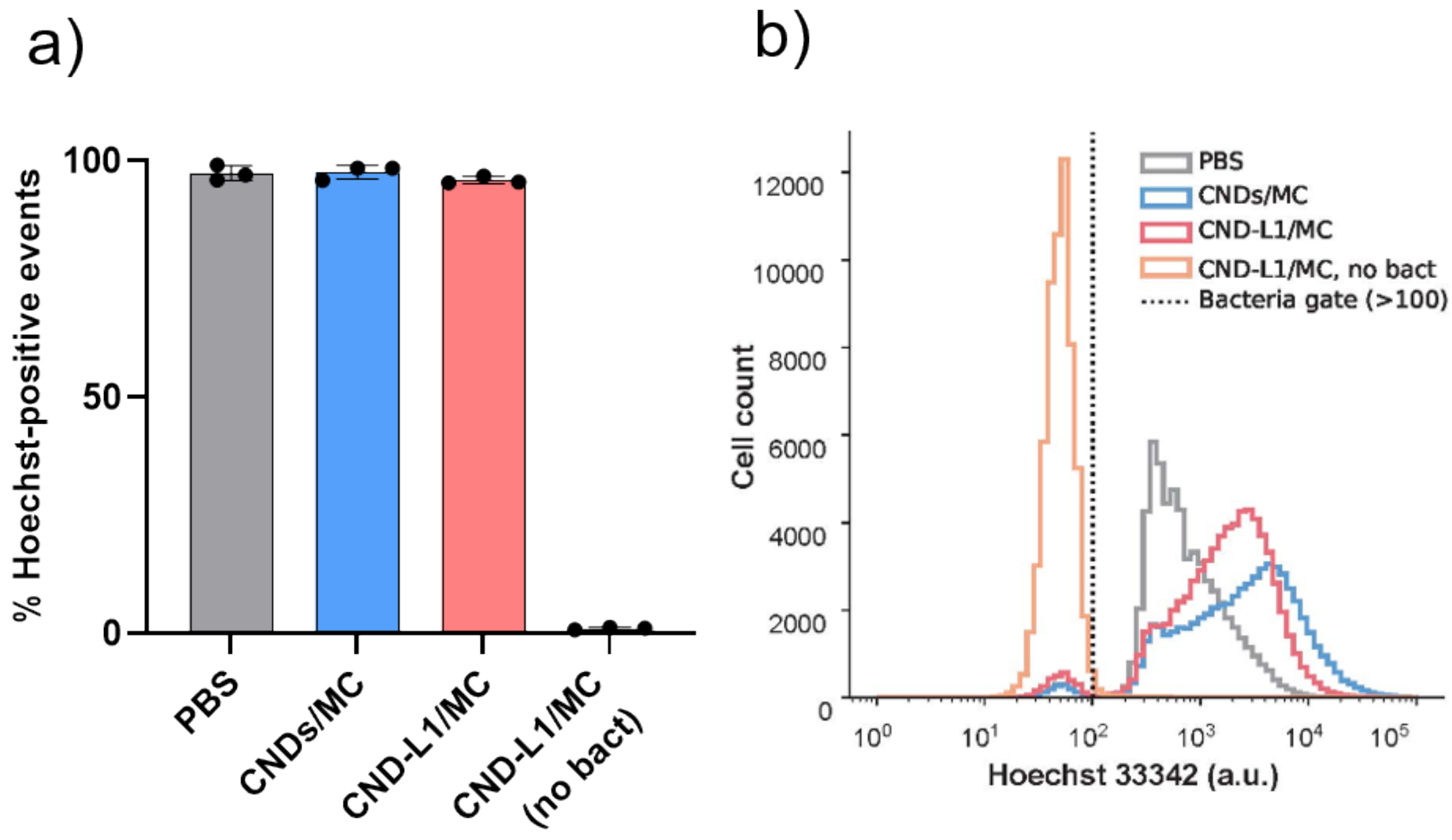

Figure S8. Validation of the Hoechst-based bacterial gating used for FACS quantification of CNDs–bacteria association. Bacteria were exposed to the samples while viable and fixed with 4% paraformaldehyde only after exposure. (a) Percentage of Hoechst-positive events (Pacific Blue-H > 100, defined as the bacterial gate) for PBS, CNDs/MC, CND-L1/MC, and the no-bacteria control (CND-L1/MC suspended in PBS without A.a.). Bars represent mean ± SD of three technical replicates. (b) Hoechst 33342 (Pacific Blue-H) fluorescence histograms for the same four samples; the dotted line indicates the bacterial gate threshold (Pacific Blue-H > 100). One representative replicate of three is shown. In the no-bacteria control, 1.2 ± 0.3% of events fell within the bacterial gate, confirming that free aggregates of the complex do not contribute appreciably to the signal measured in the samples containing bacteria.

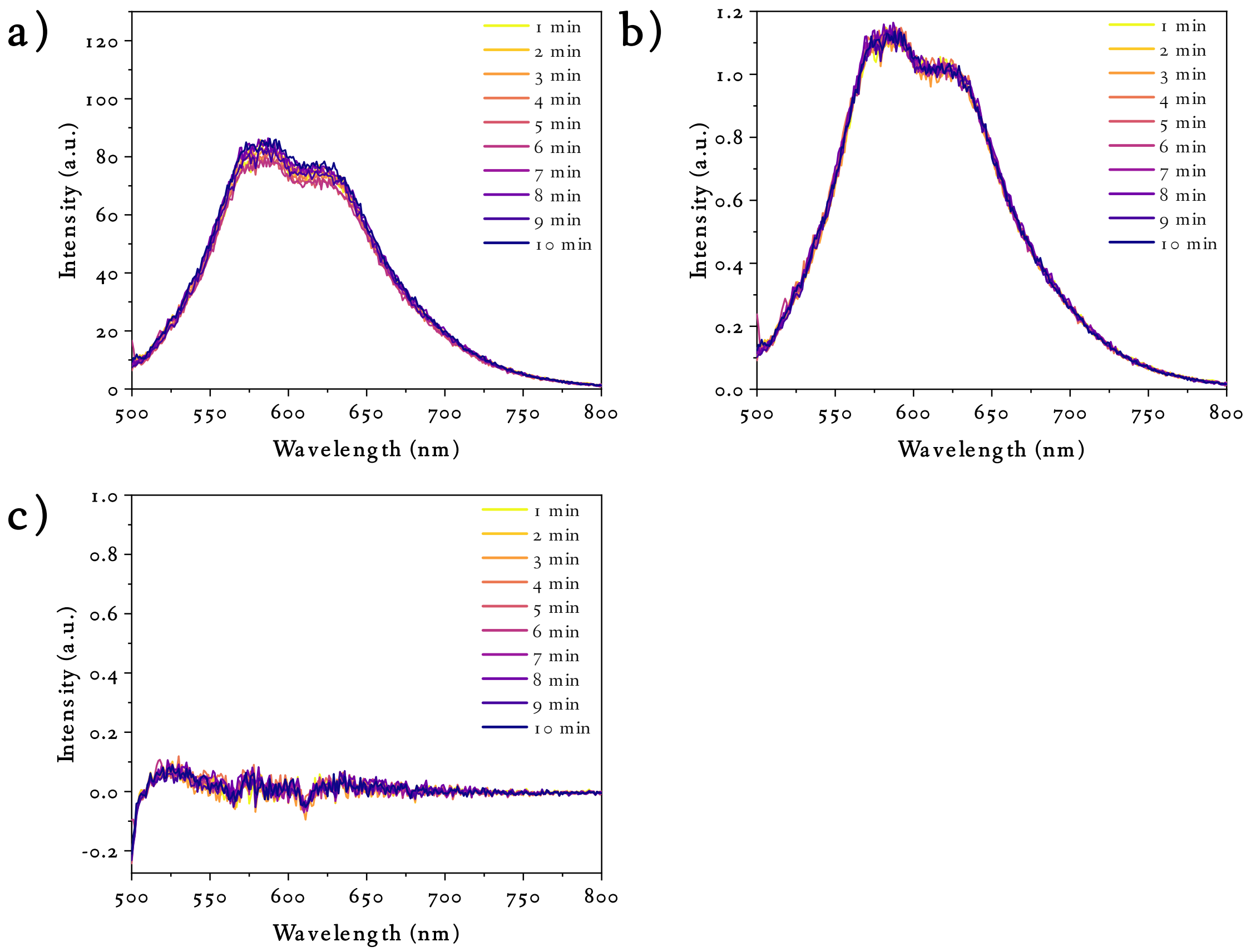

Figure S9. Emission spectra of DHR123 recorded after incubation with CNDs (25 μg mL^{-1}) in the dark for 10 min: (a) raw data; (b) spectra normalized to the CND emission at 628 nm; and (c) spectra after subtraction of the CND emission contribution.

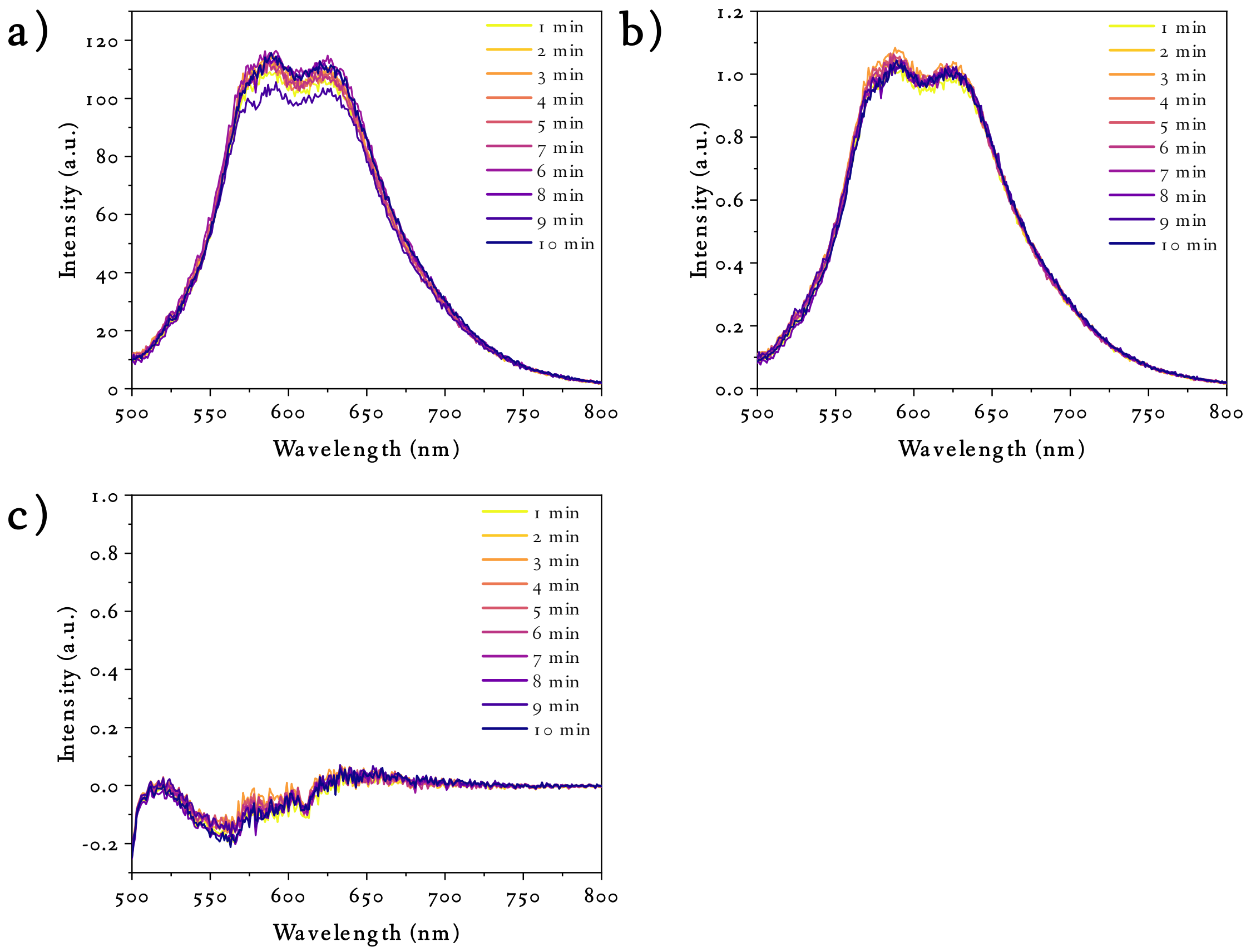

Figure S10. Emission spectra of DHR123 recorded after incubation with CNDs (25 μg mL^{-1}) following irradiation with a 660 nm laser for 10 min: (a) raw data; (b) spectra normalized to the CND emission at 628 nm; and (c) spectra after subtraction of the CND emission contribution.

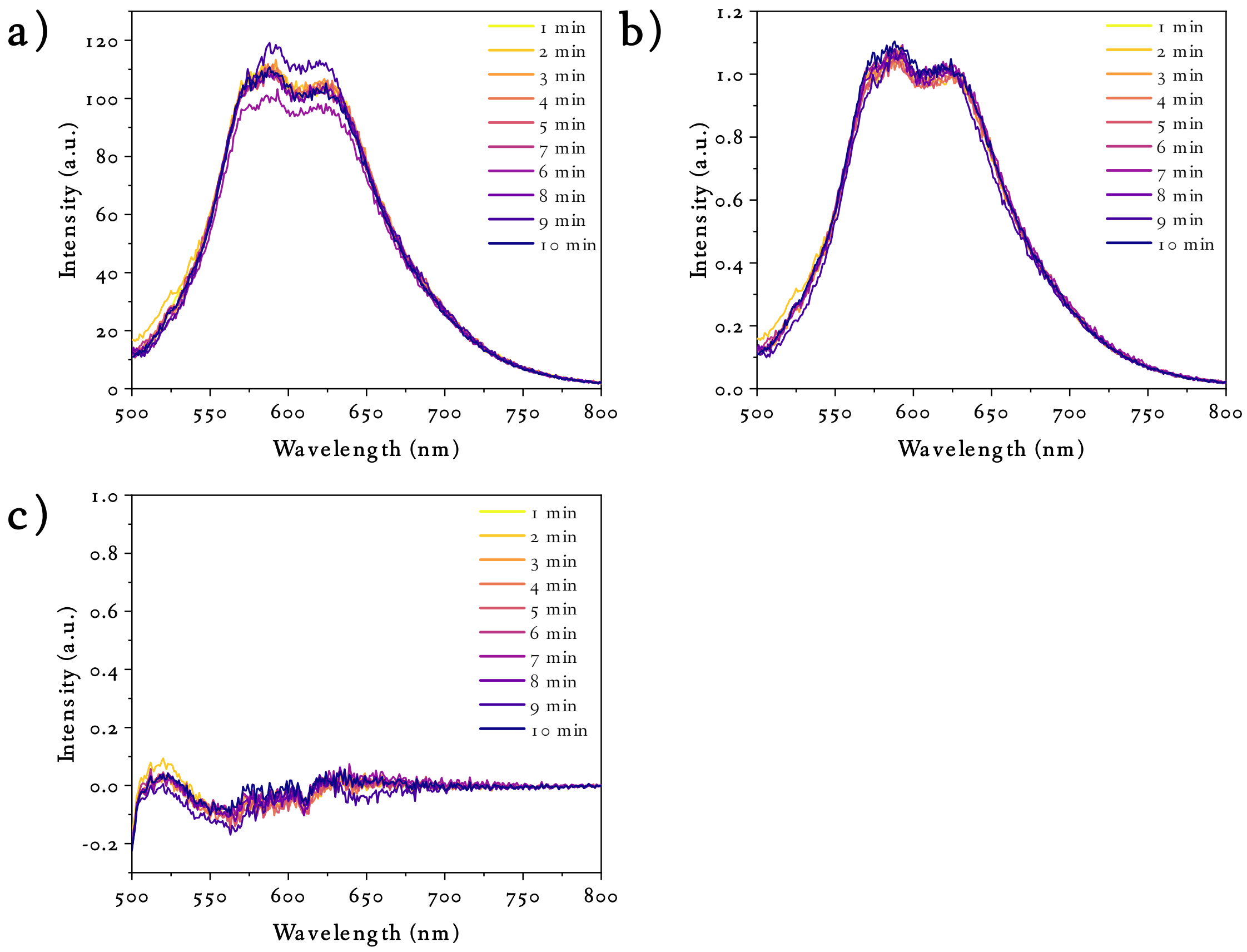

Figure S11. Emission spectra of DHR123 recorded after incubation with CNDs (25 μg mL⁻1) following irradiation with an 808 nm laser for 10 min: (a) raw data; (b) spectra normalized to the CND emission at 628 nm; and (c) spectra after subtraction of the CND emission contribution.

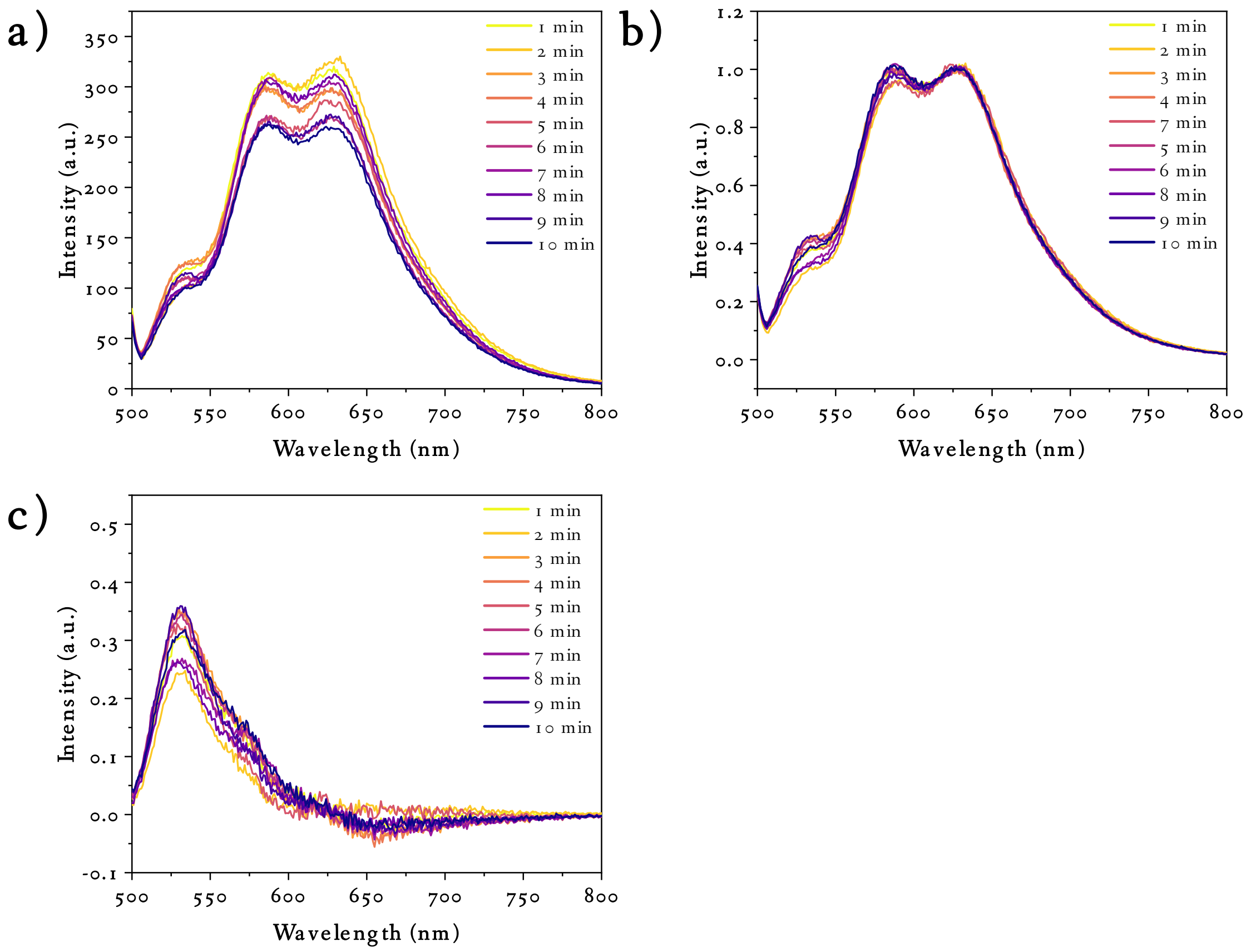

Figure S12. Emission spectra of DHR123 recorded after incubation with CND-L1 (25 $\mu g\ mL^{-1}$) in the dark for 10 min: (a) raw data; (b) spectra normalized to the CND-L1 emission at 628 nm; and (c) spectra after subtraction of the CND-L1 emission contribution.

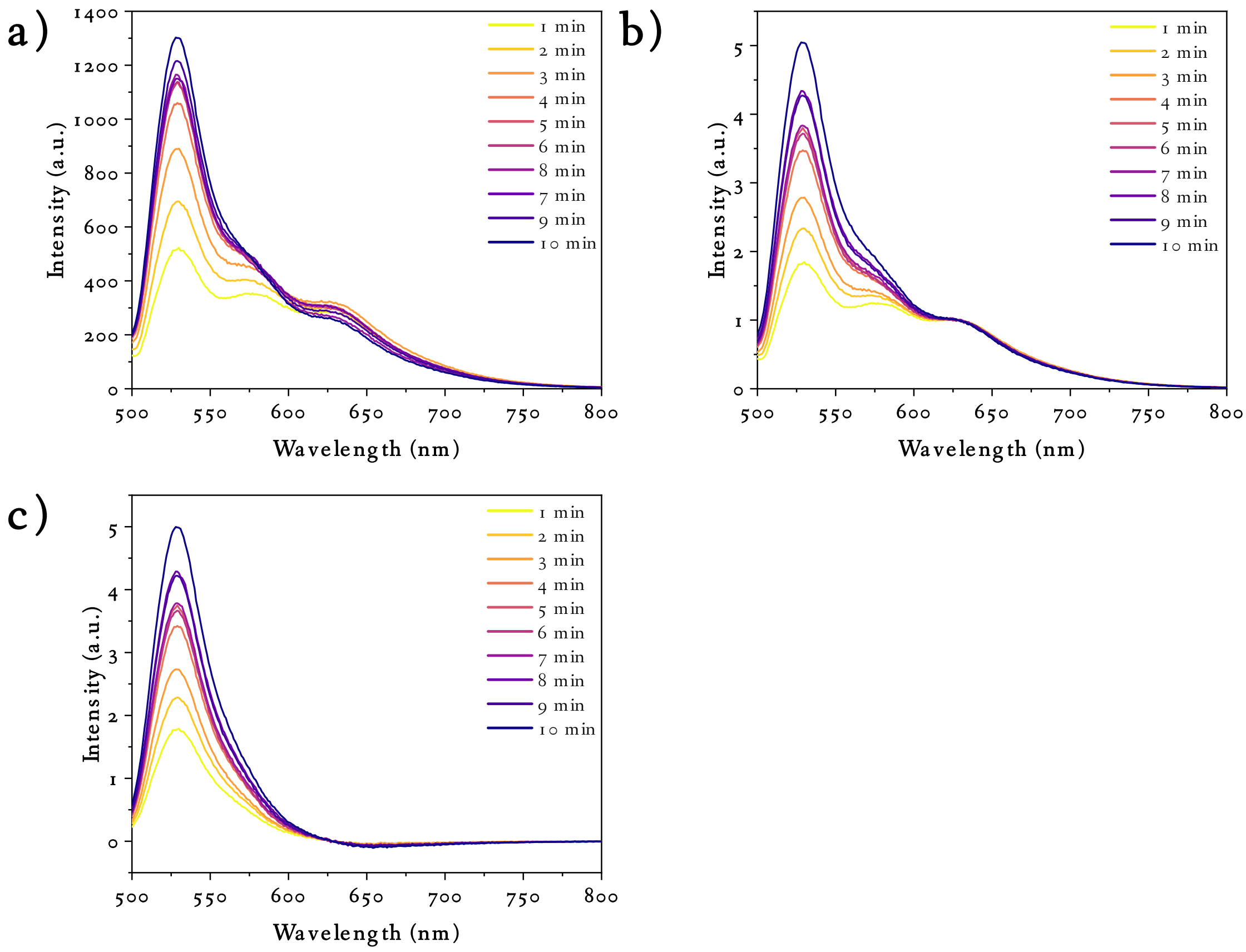

Figure S13. Emission spectra of DHR123 recorded after incubation with CND-L1 (25 μg mL^{-1}) following irradiation with a 660 nm laser for 10 min: (a) raw data; (b) spectra normalized to the CND-L1 emission at 628 nm; and (c) spectra after subtraction of the CND-L1 emission contribution.

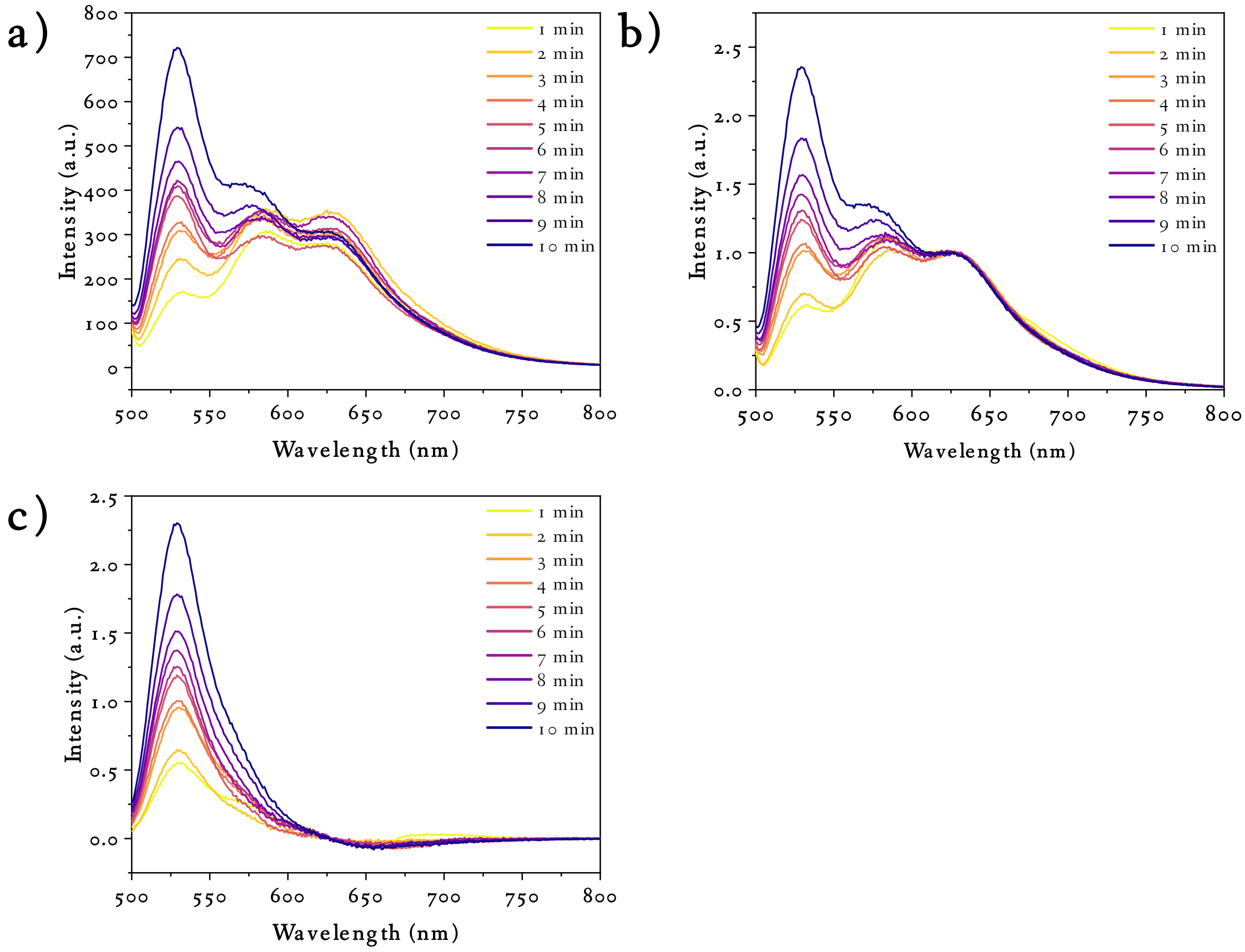

Figure S14. Emission spectra of DHR123 recorded after incubation with CND-L1 (25 μg mL^{-1}) following irradiation with an 808 nm laser for 10 min: (a) raw data; (b) spectra normalized to the CND-L1 emission at 628 nm; and (c) spectra after subtraction of the CND-L1 emission contribution.

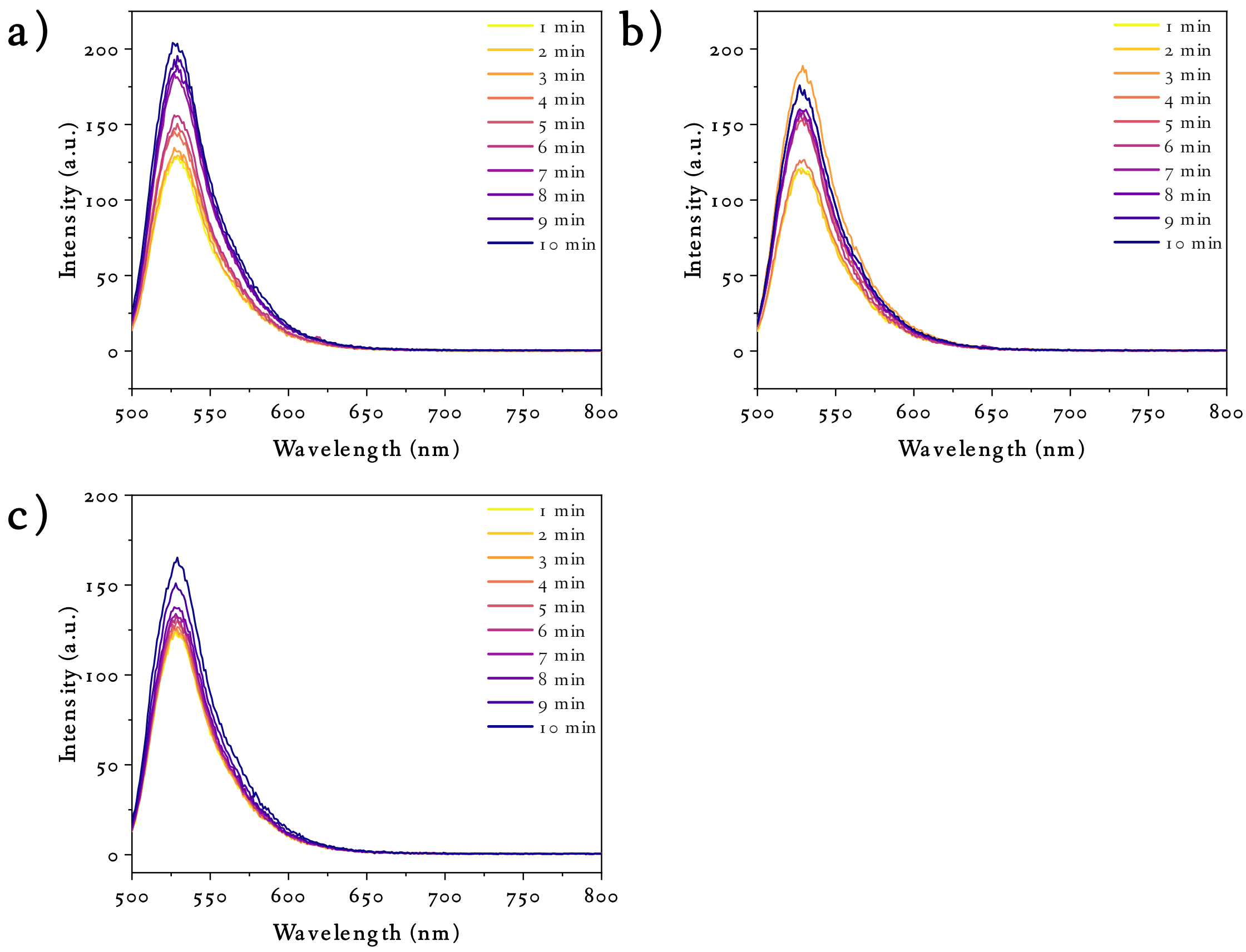

Figure S15. Emission spectra of DHR123 recorded after incubation with ligand L1 (25 μg mL^{-1}) for 10 min (a) in the dark, (b) after irradiation with a laser at 660 nm; and (c) after irradiation with a laser at 808 nm.

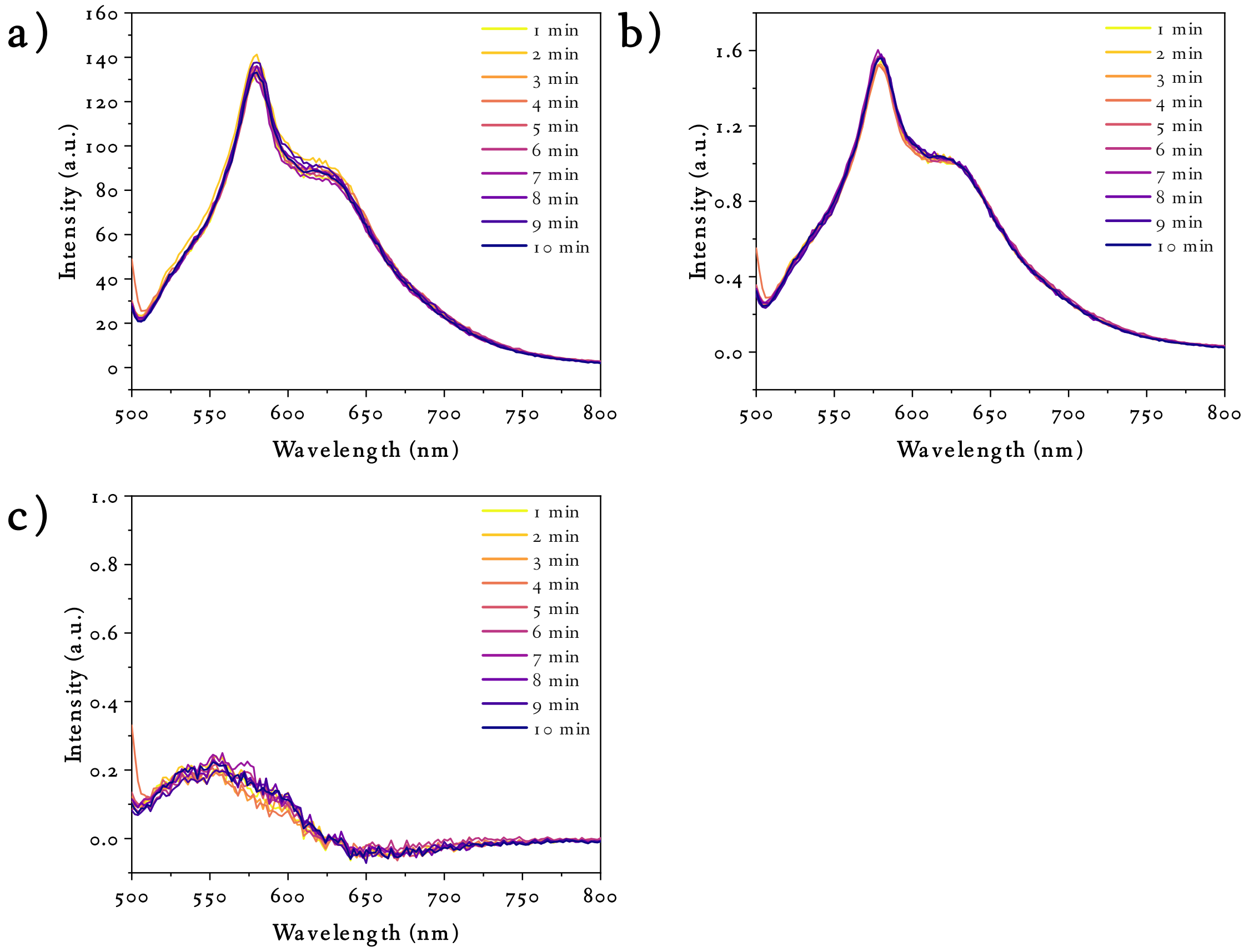

Figure S16. Emission spectra of DHR123 recorded after incubation with ligand L1 (free in solution) and CNDs (25 μg mL^{-1}) in the dark for 10 min: (a) raw data; (b) spectra normalized to the CND emission at 628 nm; and (c) spectra after subtraction of the CND emission contribution.

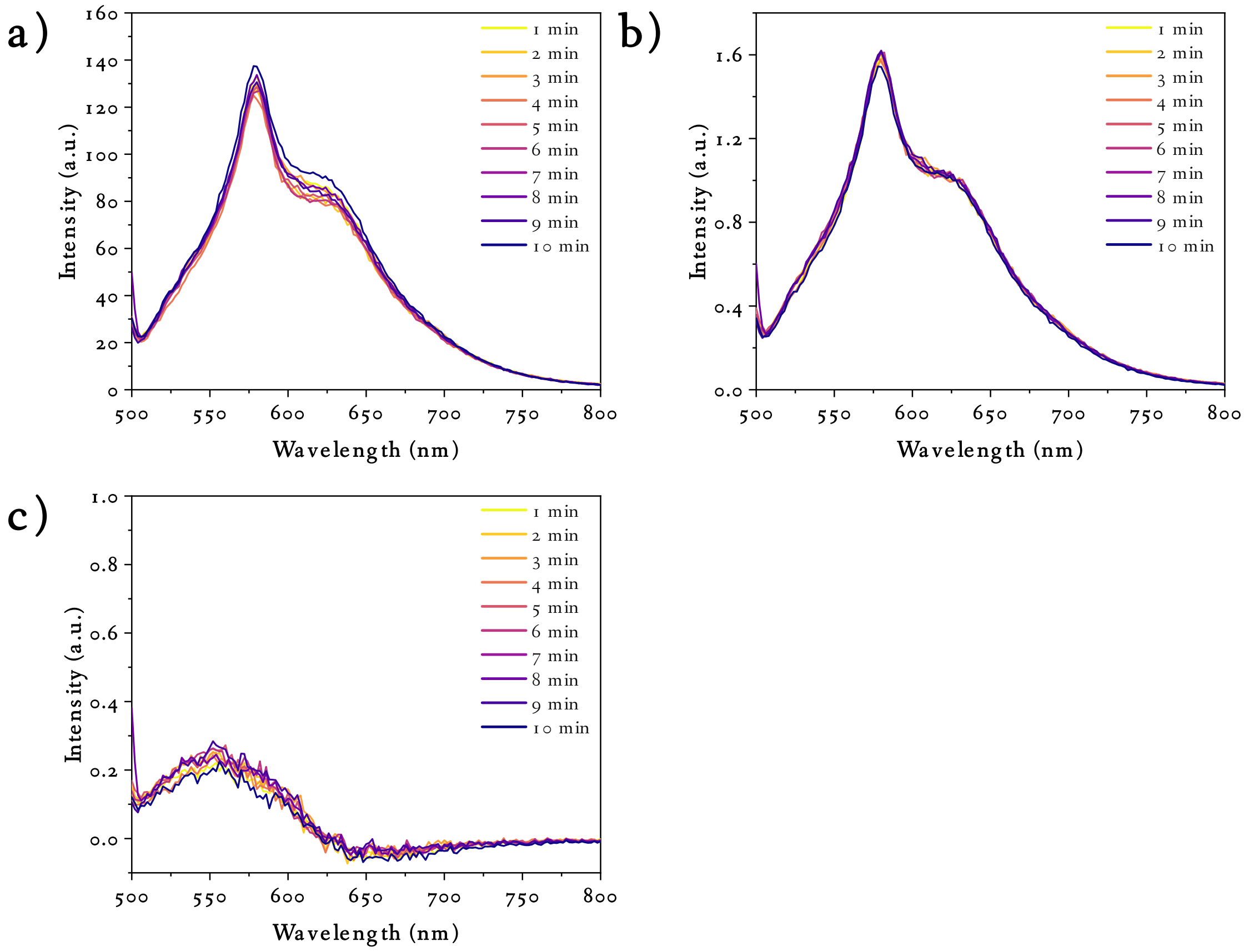

Figure S17. Emission spectra of DHR123 recorded after incubation with ligand L1 (free in solution) and CNDs (25 μg mL^{-1}) following irradiation with a laser at 660 nm for 10 min: (a) raw data; (b) spectra normalized to the CND emission at 628 nm; and (c) spectra after subtraction of the CND emission contribution.

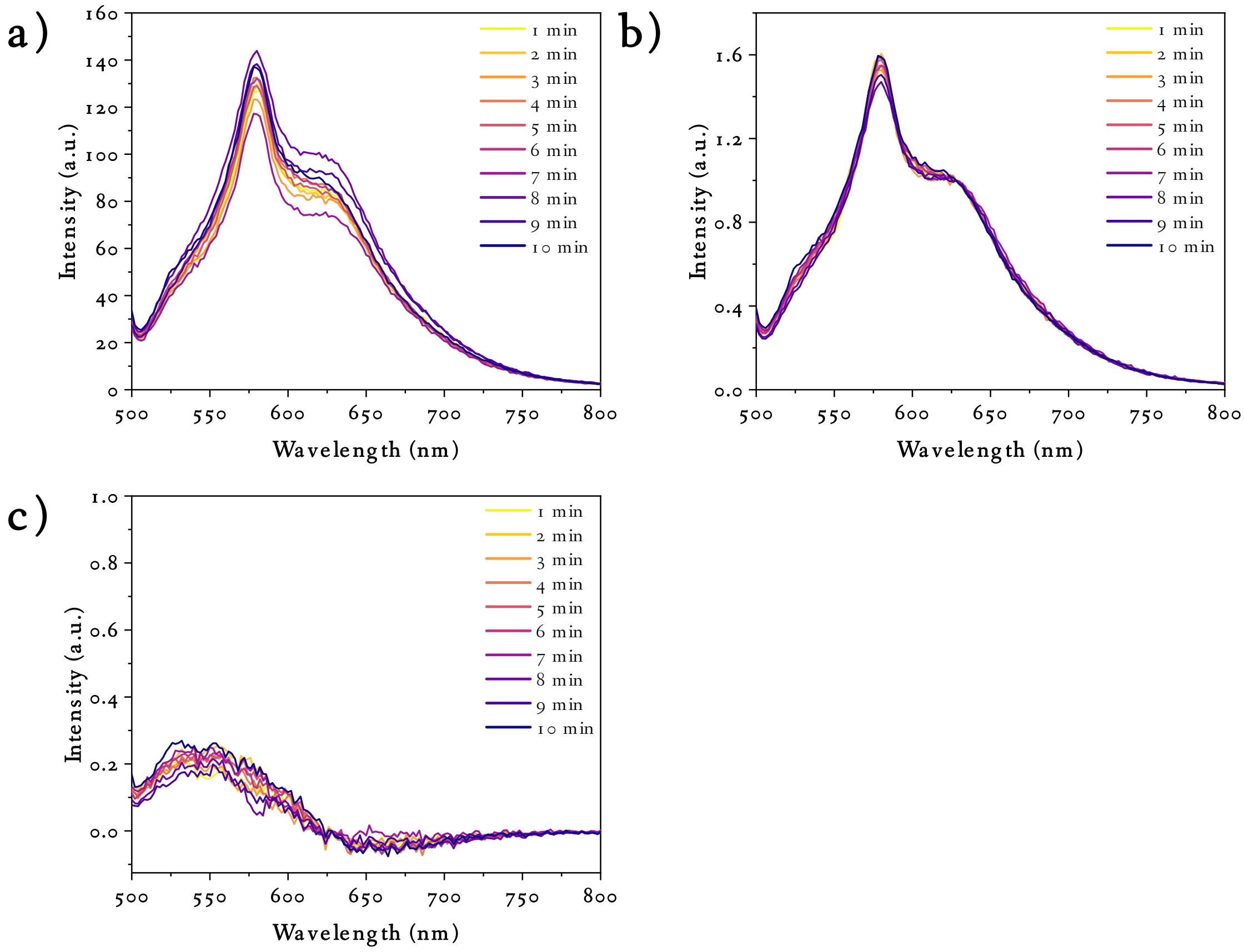

Figure S18. Emission spectra of DHR123 recorded after incubation with ligand L1 (free in solution) and CNDs (25 μg mL^{-1}) following irradiation with a laser at 808 nm for 10 min: (a) raw data; (b) spectra normalized to the CND emission at 628 nm; and (c) spectra after subtraction of the CND emission contribution.

Direct DNA interaction

To further evaluate whether direct DNA binding could contribute to the antibacterial activity, we incubated double-stranded DNA with CNDs and CND-L1, and characterized the resulting complexes by agarose gel electrophoresis and circular dichroism (CD) spectroscopy (Figure S19). Despite repeated ultrafiltration attempts with membranes of different molecular weight cut-offs, the excess CNDs could not be removed. Agarose gel electrophoresis revealed that incubation of DNA with CND-L1 produced a new band at a higher molecular weight, suggesting the formation of CND-L1/DNA multiplexes (**Figure S19a**), likely due to interactions between thymine moieties on the CND-L1 surface and the DNA helix. In contrast, DNA incubated with pristine CNDs showed no detectable change in molecular weight. CD spectroscopy further supported these observations (**Figure S19b**): DNA complexed with CNDs exhibited only a slight decrease in ellipticity compared with free DNA, whereas DNA complexed with CND-L1 showed a more pronounced decrease, consistent with conformational perturbation and the formation of higher molecular weight assemblies observed in the gel. Control CD measurements confirmed that neither CNDs nor CND-L1 alone contributed to the chirality in the measured spectral range. However, both CNDs/DNA and CND-L1/DNA solutions formed rapid macroscopic aggregates after incubation, which limited the reproducibility of the assays. Importantly, the antibacterial activity of CND-L1/MC was observed on intact bacteria where CNDs interact primarily with extracellular structures (Figure 6); direct DNA interaction is therefore unlikely to be the primary mechanism underlying the antibacterial activity at subtherapeutic MC concentrations.

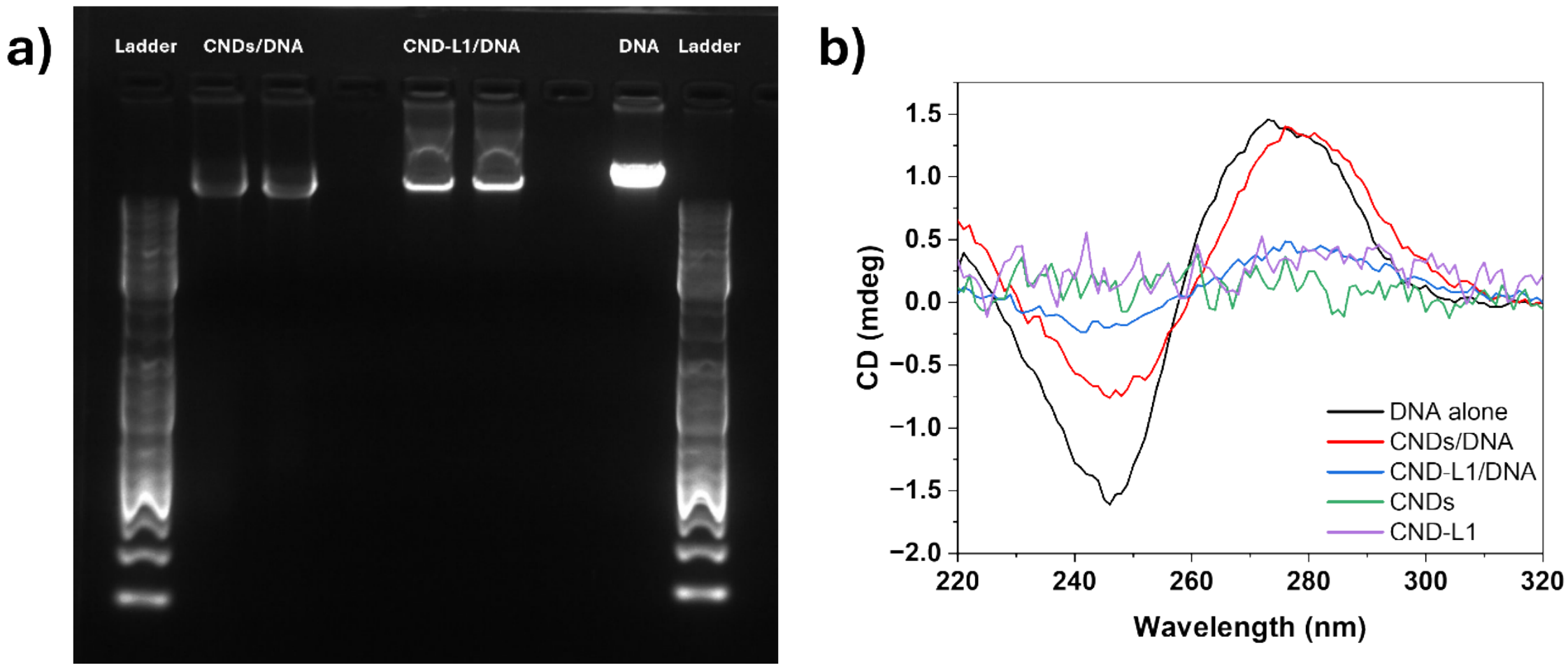

Figure S19. (a) Agarose gel electrophoresis of CNDs and CND-L1 complexed with DNA. (b) Circular dichroism spectra of CNDs, CND-L1, and their complexes with DNA.
